# Highly Oligomeric DRP1 Strategic Positioning at Mitochondria–Sarcoplasmic Reticulum Contacts in Adult Murine Heart Through ACTIN Anchoring

**DOI:** 10.3390/cells14161259

**Published:** 2025-08-14

**Authors:** Celia Fernandez-Sanz, Sergio De la Fuente, Zuzana Nichtova, Marilen Federico, Stephane Duvezin-Caubet, Sebastian Lanvermann, Hui-Ying Tsai, Yanguo Xin, Gyorgy Csordas, Wang Wang, Arnaud Mourier, Shey-Shing Sheu

**Affiliations:** 1 Center for Translational Medicine, Department of Medicine, Sidney Kimmel Medical College, Thomas Jefferson University, Philadelphia, PA 19107, USA; sergio.delafuente@uva.es (S.D.l.F.);; 2 University of Bordeaux, CNRS, IBGC, UMR 5095, F-33000 Bordeaux, France; 3 Instituto de Biología y Genética Molecular (IBGM), Departamento de Bioquímica y Biología Molecular y Fisiología, Facultad de Medicina, Universidad de Valladolid and Consejo Superior de Investigaciones Científicas (CSIC), Ramon y Cajal, 7, E-47005 Valladolid, Spain; 4 MitoCare Center for Mitochondrial Imaging Research and Diagnostics, Department of Pathology, Anatomy & Cell Biol., Thomas Jefferson University, Philadelphia, PA 19107, USA; 5 Mitochondria and Metabolism Center, Department of Anesthesiology and Pain Medicine, University of Washington, Seattle, WA 98109, USA

**Keywords:** dynamin-related protein 1 (DRP1), mitochondria-associated membranes (MAM), β-ACTIN, sarcoplasmic reticulum (SR), high Ca^2+^ microdomains, DRP1 oligomers, adult cardiomyocytes (ACM)

## Abstract

Mitochondrial fission and fusion appear to be relatively infrequent in cardiac cells compared to other cell types; however, the proteins involved in these events are highly expressed in adult cardiomyocytes (ACM). Therefore, these proteins likely have additional non-canonical roles. We have previously shown that DRP1 not only participates in mitochondrial fission processes but also regulates mitochondrial bioenergetics in cardiac tissue. However, it is still unknown where the DRP1 that does not participate in mitochondrial fission is located and what its role is at those non-fission spots. Therefore, this manuscript will clarify whether oligomeric DRP1 is located at the SR–mitochondria interface, a specific region that harbors the Ca^2+^ microdomains created by Ca^2+^ release from the SR through the RyR2. The high Ca^2+^ microdomains and the subsequent Ca^2+^ uptake by mitochondria through the mitochondrial Ca^2+^ uniporter complex (MCUC) are essential to regulate mitochondrial bioenergetics during excitation–contraction (EC) coupling. Herein, we aimed to test the hypothesis that mitochondria-bound DRP1 preferentially accumulates at the mitochondria–SR contacts to deploy its function on regulating mitochondrial bioenergetics and that this strategic position is modulated by calcium in a beat-to-beat manner. In addition, the mechanism responsible for such a biased distribution and its functional implications was investigated. High-resolution imaging approaches, cell fractionation, Western blot, 2D blue native gel electrophoresis, and immunoprecipitations were applied to both electrically paced ACM and Langendorff-perfused beating hearts to elucidate the mechanisms of the strategic DRP1 localization. Our data show that in ACM, mitochondria-bound DRP1 clusters in high molecular weight protein complexes at mitochondria-associated membrane (MAM). This clustering requires DRP1 interaction with β-ACTIN and is fortified by EC coupling-mediated Ca^2+^ transients. In ACM, DRP1 is anchored at the mitochondria–SR contacts through interactions with β-ACTIN and Ca^2+^ transients, playing a fundamental role in regulating mitochondrial physiology.

## 1. Introduction

Mitochondrial dynamics—including fission, fusion, and motility—are fundamental mechanisms in regulating mitochondrial network, morphology, and subcellular distribution [1]. Mitochondrial dynamics also control several cellular processes, including cell division, oocyte maturation [1,2], axonal projection [3,4], autophagy, and apoptosis [5,6]. Defective mitochondrial dynamics have been linked to neurodegenerative diseases, diabetes, cardiomyopathy, cancer, and several other human diseases [7,8,9]. Mitochondrial dynamics are controlled by guanosine triphosphate (GTP)-hydrolyzing proteins: dynamin-related protein 1 (DRP1) controls fission, and mitofusin 1 (MFN1), mitofusin 2 (MFN2), and optic atrophy 1 (OPA1) control fusion [10,11]. In contrast to MFN1 and MFN2, which are physically anchored to the outer mitochondrial membrane (OMM), DRP1 is a cytosolic protein. DRP1 undergoes GTP/GDP-cycle-dependent conformational changes to drive self-assembly and disassembly, but the structural bases of this process are still incompletely understood [12].

DRP1 is detected ubiquitously in mammalian tissues, with the highest levels found in energy-demanding tissues like the heart, skeletal muscles, and brain [13,14]. The high level of DRP1 in the heart seems to be at odds with numerous studies showing that mitochondrial dynamics in adult ventricular myocytes occur rather infrequently compared to other cell types [15,16,17,18,19]. Despite the scarcity of mitochondrial dynamics in adult cardiac mitochondria [20], DRP1 is essential for controlling several functions critical in heart development, suggesting a possible non-canonical role of DRP1 in the cardiac tissue [20,21,22,23,24].

We recently reported that inhibition of DRP1 activity pharmacologically or genetically resulted in only a mild change in mitochondrial morphology in face of a significant decrease in State 3 respiration in adult cardiomyocytes (ACM) [25]. These results uncovered a novel non-canonical function of DRP1 in positively stimulating mitochondrial bioenergetics in ACM, which is likely independent of mitochondrial fission. In human osteosarcoma cells (U2OS), DRP1 is in a dynamic equilibrium of binding and release from mitochondria, with only a low percentage of oligomerized DRP1 puncta (2.5% of total puncta) engaged in observable fission events during the 10 min imaging period [26,27]. This process of DRP1 assembly and oligomerization on mitochondria is called maturation. Specific signals—including actin filament and cytosolic Ca^2+^—play a critical role in DRP1 maturation. The authors proposed that these DRP1 puncta travel along the OMM in search of fission signals to readily execute their canonical function. However, it is also plausible that some of these mitochondria-bound DRP1 oligomers may have non-canonical functions that are independent of fission. Knowing the existence of this DRP1 non-canonical role, the most immediate question is to elucidate where and how DRP1 is specifically located within the adult mouse cardiomyocytes and what function there.

Mitochondria occupy ~35% of the cell volume in the heart [28]. The majority of these mitochondria appear fragmented, show little mobility, and are located between the myofibers, with a significant number tethered to the junctional sarcoplasmic reticulum (jSR) near the dyads [29]. The juxtaposition of mitochondria to the jSR leads to the formation of a high Ca^2+^ microdomain when Ca^2+^ is released from the SR during excitation–contraction (EC) coupling [29]. This high Ca^2+^ microdomain provides privileged Ca^2+^ transport from the jSR to the mitochondria through the mitochondrial Ca^2+^ uniporter complex (MCUC). It is widely believed that mitochondrial Ca^2+^ uptake through MCUC stimulates adenosine triphosphate (ATP) regeneration processes rapidly and robustly to meet the extremely high energy requirements for perpetual blood pumping, known as the excitation–bioenergetics (EB) coupling [30]. However, this concept has been challenged by recent findings showing that MCU knock-out causes little energy crisis in the beating heart [31,32,33].

We and others have shown that increases in cytosolic Ca^2+^ concentration ([Ca^2+^]_c_) promote DRP1 translocation from cytosol to mitochondria in cardiomyocytes [34]. Based upon these reported data, we hypothesize that in ACM, oligomeric DRP1 is strategically localized at the mitochondria–jSR contacts where high [Ca^2+^] microdomains are created during EC coupling. Such localization would enhance the sarcoplasmic reticulum–mitochondria (SR–mito) communication, favoring the mitochondrial bioenergetics control. To test this hypothesis, we have applied multiple techniques encompassing Airyscan confocal imaging, immunogold electron microscopy, subcellular fractionations, and blue native polyacrylamide gel electrophoresis (BN-PAGE) [16,35,36].

The results showed, for the first time, that in the heart, the mitochondrial membrane-bound DRP1 localized in high molecular weight clusters at the mitochondria–SR interface, with surprisingly no detectable DRP1 in the highly pure mitochondria fraction. This preferential accumulation was preserved by mitochondria-associated β-ACTIN and EC coupling-linked Ca^2+^ transients. This unique feature of DRP1 oligomers positioning at the high Ca^2+^ functional microdomains between mitochondria and SR could enable its non-canonical role in regulating heart functions.

## 2. Materials and Methods

### 2.1. Animals

Experimental preparations were derived from the hearts of 12- to 14-week-old male C57BL/6 mice and male Sprague–Dawley rats. Both male and female rodents were initially tested, and no significant sex differences were observed in the measured outcomes, including DRP1 localization among the fractions and DRP1 depletion in the DRP1cKO. Therefore, to reduce variability and maintain consistency in the study, subsequent experiments were conducted using only male mice/rats. This approach allowed for more precise analyses while minimizing unnecessary animal use, in line with the principles of the 3Rs (reduction, refinement, and replacement) in animal research. The DRP1icKO was generated by crossing the DRP1fl/fl mouse (obtained from Dr. Junichi Sadoshima, Rutgers New Jersey Medical School) with the aMHC-Mer-Cre-Mer (Cre+) mouse line (JAX). Cre-mediated DRP1 ablation was induced by tamoxifen IP injection (40 mg/kg/day) for 3 consecutive days. Experiments were carried out 6 weeks after the first injection. Animal handling was done in accordance with the National Institutes of Health Guide for the care and use of laboratory animals, and the protocols were applied in compliance with the Thomas Jefferson University IACUC guidelines. Animal handling and experimental procedures were conducted in full compliance with the guidelines of the Ministère Français de l’Éducation Nationale et de l’Enseignement Supérieur et de la Recherche. All procedures conducted at the Animal House A2 were validated by the Ethics Committee N°050 and officially approved by the French Ministry of National Education and Research (APAFIS#12648-2017112083056692v7).

### 2.2. Isolation of Mouse Cardiomyocytes

To obtain ventricular adult cardiomyocytes (adult cardiomyocytes from this point), mice were sacrificed by cervical dislocation and anticoagulated using an intraperitoneal (IP) heparin (50 IU/mice) injection. Hearts were retroperfused through the aorta in a Langendorff system (Radnoti, Covina, CA, USA) according to the protocol previously described [37,38]. Freshly isolated adult cardiomyocytes were used in all experimental procedures.

### 2.3. Electrical Field Stimulation on Isolated Adult Mouse Cardiomyocytes

The electrical field stimulation experiments performed to elucidate DRP1 localization in adult cardiomyocytes were done as previously described [37,39]. Briefly, isolated calcium-tolerant cardiomyocytes from adult hearts were placed in 25 mm diameter coverslips pretreated with laminin and mounted in an electrical stimulation chamber equipped with two platinum electrodes (RC-47FSLP, Warner Instruments, Hamden, CT, USA). Cells were continuously perfused with field stimulation buffer that contained in mM: 150 NaCl, 5.4 KCl, 10 HEPES acid, 1 glucose, 2 pyruvate, 5 creatine, and 5 taurine, supplemented with 2.5 μM or 2 mM CaCl_2_. Electrical biphasic pulses of 25–35 V amplitude, 2 Hz frequency, and 5 ms duration were applied to the cells for 15 min (MyoPacer Stimulator, IonOptix, Westwood, MA, USA). Then, cardiomyocytes were fixed and subjected to a standard immunofluorescence (IF) staining protocol for further analysis.

### 2.4. Isolation of Subcellular Fractions from Murine Heart

Cytosol (Cyt), SR, crude mitochondria (cMit), pure mitochondria (pMit), and mitochondria-associated membranes (MAM) enriched fractions were obtained from murine heart homogenates by fractionation as described before [39]. Briefly, fresh mouse ventricles were minced and homogenized using a Potter–Elvehjem PTFE pestle-glass tube (Fisher Scientific GmbH, Schwerte, Germany) (loose followed by tight) on ice-cold isolation buffer containing (mM): 10 HEPES acid, 225 mannitol, 75 sucrose, 0.1 EGTA, at pH = 7.4. After an initial centrifugation step (750× *g* for 5 min, 4 °C), the supernatant was again centrifuged (7000× *g* for 10 min, 4 °C). The resulting pellet contained cMit fraction. The supernatant was centrifuged (40,000× *g* for 45 min, 4 °C) to obtain the SR-enriched fraction in the pellet. This pellet consists of SR that also contains small functional mitochondrial particles that remain attached to it, as described before [37]. This fraction will be defined throughout this paper as SR. The supernatant was further ultracentrifuged (100,000× *g* for 1 h, 4 °C) to obtain the cytosolic fraction in the supernatant. cMit fraction previously obtained was further purified in a 30% Percoll gradient ultracentrifuged (65,000× *g* for 30 min, 4 °C) in a swing-out rotor, resulting in pure mitochondria (heavy fraction) and MAM (light fraction). Supplementary Figure S1 shows a detailed schematic representation of the cardiac cellular fractions. The high content of RyR2 and SERCA in the MAM fraction confirmed the extraction of mitochondria-associated SR membrane.

### 2.5. Protein Analysis and Western Blot

Equal amounts of protein (70 μg) from the subcellular fractions supplemented with 5× protein loading buffer (National Diagnostics, Atlanta, GA, USA) were preheated (95 °C, 5 min), separated on 12% acrylamide/bis (Bio-Rad, Hercules, CA, USA) gels, and transferred to Amersham Hybond–ECL nitrocellulose membranes, pore size 0.2 µm (GE Healthcare, Chicago, IL, USA). After blocking with Odyssey™ blocking buffer (PBS) for 1 h at room temperature, the membranes were incubated with the primary antibodies of interest—DRP1 (BD; 61113), β-ACTIN (Santa Cruz Biotechnology Inc., Dallas, TX, USA; sc-4778), SERCA2 (Thermo Fisher, Waltham, MA, USA; MA3-919), HSP60 (Cell Signaling Technology, Beverly, MA, USA; 12165), ANT (Mitosciences, Eugene, OR, USA; MSA02), TOM20 (Santa Cruz Biotechnology Inc.; sc-11415), calsequestrin (Abcam, Cambridge, MA, USA; ab3516), FIS1 (ENZO, Farmingdale, NY, USA; ALX-210), MiD49 (Proteintech; 16413-1-AP), MiD51 (Proteintech, Rosemont, IL, USA; 20164-1-AP), MFF (Abcam; ab129075), INF-2 (GeneTex, Irvine, CA, USA; CTX130714), SPIRE-1C (custom-made, obtained from Dr. Uri Manor, Salk Institute, La Jolla, CA, USA), TIM23 (BD; 611222), and GAPDH (Cell Signaling Technology; 2118L)—in Odyssey™ blocking buffer (PBS) overnight at 4 °C. Infrared secondary antibodies—IRDye 680LT goat anti-mouse and IRDye 800CW goat anti-rabbit (LI-COR Biosciences, Lincoln, NE, USA) and/or horseradish peroxidase (HRP)-conjugated goat-anti-mouse and goat-anti-rabbit antibodies (Cell Signaling Technology)—were used for visualization of the proteins by LI-COR Odyssey scanner (LI-COR Biosciences). The specificity of the DRP1 antibody was checked for WB in isolated cardiomyocyte lysate from DRP1icKO (Figure S2A, right panel). Image Studio™ Lite software 5.0 (LI-COR Biosciences) was used for band quantification and densitometry analysis.

### 2.6. First Dimension Light Blue Native Polyacrylamide Gel Electrophoresis (LBN-PAGE)

For LBN-PAGE, 100–300 μg of protein from each fraction of study were treated with extraction buffer pH 7.4 (10 μL/100 μg) containing (mM): 30 HEPES, 150 CH3CO2K, 2 ε-amino-n-caproic acid, 1 EDTA, and 12% (*v*/*v*) glycerol supplemented with 3% (*w*/*v*) digitonin (Sigma Aldrich, Saint Louis, MO, USA) for 30 min at 4 °C. Extracts were mixed with loading dye (1/400 of 5% [*w*/*v*]) Coomassie Brilliant Blue G-250 (Sigma Aldrich) (1 ul loading dye per 30 ul extraction) and 750 mM ε-amino-n-caproic acid at pH 7.0. Native PAGE Novex 3–12% Bis-Tris Protein Gels (Life Technologies, Carlsbad, CA, USA) were loaded with 100–300 μg extracts. After electrophoresis, lanes were stained with Coomassie Brilliant Blue R-250 or used for second-dimension analysis.

### 2.7. Second-Dimension SDS-PAGE

Lanes from the first-dimension light blue native gels were first treated with equilibration buffer containing (mM): 6000 urea, 50 Tris-HCl, 30% glycerol, and 2% SDS for 5 min. On a second step, lanes were treated with equilibration buffer supplemented with 100 mM DTT (Sigma Aldrich) for 30 min, followed by treating with equilibration buffer supplemented with 135 mM Iodoacetamide (Sigma Aldrich) for 15 min. The remaining DTT and iodoacetamide were removed by a final washing step with equilibration buffer for 15 min.

Treated lanes were mounted on 4–12% Bis-Tris NuPAGE™ Protein Gels (Novex™ Invitrogen™, Thermo Fisher Scientific, Waltham, MA, USA) and transferred to Amersham Hybond–ECL nitrocellulose membranes (GE Healthcare). The membranes were blocked in TBS-T solution (Tris-buffered saline, 0.1% Tween-20) with 5% non-fat milk powder. Proteins were detected with DRP1 in TBS-T with 3% non-fat milk powder. HRP-conjugated IgG anti-mouse antibodies (Cell Signaling Technology) were used as secondary antibodies. Peroxidase reactions were carried out and visualized using Supersignal West Dura Extended Duration Substrate (Thermo Fisher Scientific) and the chemiluminescence imaging system GBOX-Chemi-XRQ gel documentation system, Syngene. Image Studio™ Lite software (LI-COR Biosciences) was used for band quantification and densitometry analysis.

### 2.8. Immunofluorescence (IF) and Imaging Analysis

Isolated cardiomyocytes were plated on laminin-coated coverslips and fixed (4% PFA). A 3% BSA-0.2% Triton^®^X-100 solution was used for permeabilization and blocking, followed by a 1 h RT incubation with PBS–1% BSA containing mouse monoclonal DRP1 antibody (611113, BD Transduction Laboratories, San Jose, CA, USA; 1:50) followed by a 1 h RT incubation with PBS–1%BSA containing rabbit polyclonal RyR2 (PA5-36121, Thermo Fisher, 1:50) and/or rabbit polyclonal TOM20 antibody (Santa Cruz, 1:50). Alexa Fluor^®^488 or 647 were used as secondary antibodies. SlowFade^®^ was used for mounting on microscope slides. Specificity of the DRP1 antibody was checked for IF in DRP1KO–mouse embryonic fibroblast (MEF) cell line, adult cardiomyocytes from DRP1ciKO mice, and rat adult cardiomyocytes treated with DRP1shRNA (Figure S2A–C).

Immunofluorescence was imaged using the Zeiss LSM880 (Zeiss, Osseo, MN, USA) system equipped with the Airyscan super-resolution (1.7x beyond diffraction limit) detection system and ZEN software. A 63X Zeiss plan-apochromat oil, 1.4 NA, DIC lens (Zeiss) was used to obtain all images. Image analyses were done using FIJI/ImageJ (version 2.0.0, 2.1.0, NIH). DRP1 particles are likely representing oligomerized DRP1 because the fluorescent particles with lower intensity were removed through thresholding.

For the RyR2/TOM20-DRP1 images, the same threshold was applied to each channel. Specifically, for the DRP1-immunolabeled images, a threshold considering only 5% of the maximal intensity was applied. With this setting, only the brighter signals were considered, which is coincident with the DRP1 oligomerization (puncta pattern). By using this mask, the resulting coincident particles from the two channels with >0.05 μm^2^ area were counted. In a second step, the RyR2 signal was dilated by 66 nm and 132 nm (1 and 2 pixels) from the original labeling. By using this new mask, the same particle coincidence analysis strategy was applied. A total of 5 to 10 microscope fields of 1275 µm^2^ were analyzed for each condition.

For the TOM20–DRP1 images, the same threshold was applied to each channel. By applying the same analysis strategy, the DRP1 particles found in contact with the outer mitochondria membrane (OMM) at different distances were counted. In a second step, the particles were classified by their localization at the transversal side (T side) or longitudinal side (L side) of the mitochondria for each condition. Supplementary (Figure S3), shows a detailed example of this analysis.

### 2.9. Pre-Embedding Immunogold (IG) and Transmission Electron Microscopy (TEM)

Isolated mouse ventricular cardiomyocytes were fixed with 5% paraformaldehyde/PBS, followed by quenching with glycine and permeabilization and first blocking with 3% BSA+0.2% Triton-X. Cardiomyocytes were incubated for 1 h in primary antibody diluted in 1% BSA (1:100) + 0.2% Triton-X, followed by secondary blocking with 5% goat serum in PBS. Secondary antibody FluoroNanogold™ (anti-mouse, 1:100) was diluted in 1% goat serum and 0.1% TritonX-100 (1 h incubation). As control for non-specific binding, IG-TEM was performed in the absence of primary antibody, using the above-mentioned secondary antibody labeling (Figure S1C). Nanogold particles were enhanced for 3 min using GoldEnhance™ (Nanoprobes, Yaphank, NY, USA). Gold enhancement was followed by fixation with 1.6% glutaraldehyde and 0.2% tannic acid in PBS; post-fixation with 1% osmium tetroxide in 0.1 M sodium cacodylate buffer; and contrasting with 1% aqueous uranyl acetate. The cells were dehydrated in graded alcohol and embedded in Durcupan. Upon polymerization, the coverglass was dissolved using concentrated hydrofluoric acid, after which ultrathin sections (65–80 nm) were prepared using a Leica UTC ultramicrotome (with Diatome diamond knife) (Leica Camera, Teaneck, NJ, USA) and imaged with a Thermo/FEI Tecnai 12 120 keV transmission electron microscope (Thermo Fisher Scientific, Hillsboro, OR, USA). Images were taken at 3200–15,000× direct magnification (at 80 keV). Analysis of gold particles was performed using FIJI/ImageJ (NIH).

Mitochondrial morphology assessment was performed as described before [40]. For the analysis of the DRP1–particle distribution around intermyofibrillar mitochondria, the T (transversal) and L (longitudinal) sides were considered as the minor and major axes, respectively, and compared with the total perimeter of the organelle (S4).

### 2.10. Langendorff Perfusion of Rat Hearts

Adult male Sprague–Dawley rats (250–300 g) were anesthetized with isoflurane (3% in oxygen) and submitted to a bilateral thoracotomy. Whole hearts were quickly excised and retrogradely perfused through the aorta with an oxygenated (95% O_2_–5% CO_2_) Krebs solution at 37 °C in mM: 118 NaCl, 4.7 KCl, 1.2 MgSO_4_, 25 NaHCO_3_, 1.2 KH_2_PO_4_, and 11 glucose at pH 7.4 in a constant-flow Langendorff system, as previously described [41]. After 15 min of equilibration, two hearts were treated in normoxic conditions with low or physiological concentrations of Ca^2+^ (2.5 µM or 1.8 mM, respectively). Each concentration was applied for 60 min. After the treatments, hearts were collected, and different cellular fractions were obtained, as described above.

### 2.11. Citrate Cynthase Activity Measurements

Citrate synthase activity was determined by colorimetry and was used to normalize data on mitochondrial respiration [42].

### 2.12. Statistical Analysis

Data are expressed as mean ± SEM, and two-tailed Student’s t-test was used for comparisons between two independent sample groups. Factorial nested ANOVA analysis was applied for comparison of groups with more than one factor, by a priori comparison when necessary. Differences of *p* < 0.05 were considered statistically significant (two-tailed significance was commonly applied and one-tailed when justified by previous data and predictions about the direction of the difference). When samples did not follow a normal distribution, the non-parametric test of the median was used. All statistical analyses were performed with IBM-SPSS v.24 software (New York, NY, USA).

## 3. Results

### 3.1. DRP1 Is Preferentially Located at the Mitochondria–jSR Contacts in Primary Adult Cardiomyocytes

**Figure 1:**
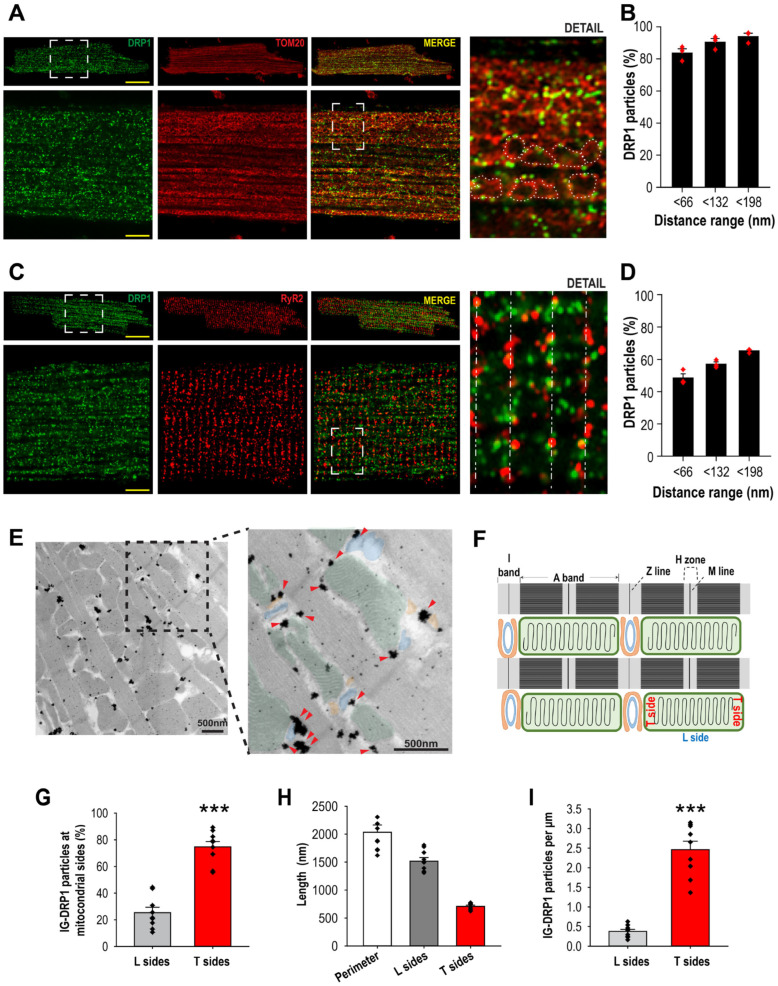
DRP1 is preferentially located at SR–mitochondria contacts in adult cardiomyocytes. (**A**) Top panels, confocal images showing immunostaining of DRP1 (green), TOM20 (red), and merging of the two images in ACM (scale bar = 20 mm). Lower panels: 4× zoomed images adapted from the dashed line box after AiryScan processing (scale bar = 5 mm). “Detail” zoomed image adapted from the dashed line bracket in the adjacent panel demonstrates the proximity of DRP1 puncta with mitochondrial OMM (encircled by the white dots). (**B**) Summarized data showing the fraction of DRP1 within the indicated distance ranges (<66, <132, and <198 nm) from TOM20. N = 3 animals, 7–9 cells per animal. (**C**) Top panels, confocal images showing immunostaining of DRP1 (green) and RyR2 (red) in an ACM (scale bar = 20 mm). Lower panels: zoomed images are after AiryScan processing (scale bar = 5 mm). “Detail” zoomed image at far-right panel shows the proximity of DRP1 with RyR2 along the Z-lines (dashed line). (**D**) Summarized data showing the fraction of DRP1 within the indicated distance ranges (<66, <132, and <198 nm) from RyR2. N = 3 animals, 7–9 cells per animal. (**E**) Immunogold TEM detection of DRP1 in ACM. Electron microscopy of DRP1 labeled with immunogold (IG). Zoom: colored labels highlight SR (orange), T-tubules (blue), and mitochondria (green). Red arrows indicate DRP1 particles. (**F**) For quantitative analysis of the location of DRP1–IG particles, a schematic diagram is generated to illustrate the transversal (T) side of mitochondria, where jSR, T-tubule, and mitochondria are in juxtaposition, and longitudinal (L) side of mitochondria, where no jSR is in proximity. (**G**) Summarized data showing the percentage of DRP1–IG positive particles associated with the L or T side of the mitochondria. (**H**) The lengths of the mitochondrial perimeter, L sides and T sides (nm). N = 4 cells, 2–4 fields per cell, 122 mitochondria, ***: *p* < 0.001. (**I**) Representation of the IG-DRP1 particle per µm was obtained by normalizing the number of particles per side (T or L) by the length of each side (µm). N = 4 cells, 2–4 fields per cell, total of 115 mitochondria, ***: *p* < 0.001. Two-tailed Student’s t-test was used for comparisons between two independent sample groups.

To test our hypothesis that DRP1 preferentially accumulates at mitochondria–jSR contacts, we first characterized the subcellular distribution of DRP1 by using imaging approaches. Initially, we used the Airyscan (Zeiss LSM880) super-resolution imaging system to quantitatively evaluate DRP1 location via immunofluorescence (IF) staining. We labeled mitochondria with TOM20 and jSR with RyR2 (Figure 1A,C). We quantified DRP1 colocalization with either TOM20 or RyR2 (Figure 1A,C) by calculating the proportion of DRP1 puncta located within increasing distance thresholds (<66, <132, and <198 nm) from each respective mask (see Methods [37]). Quantitative analysis showed that 83.76 ± 2.60% of the DRP1 particles overlapped with TOM20 (distance <66 nm), confirming the predominant mitochondrial association of DRP1, and 93.95 ± 2.06% were located within the next adjacent pixel (<132 nm) (Figure 1B). These results were consistent with previous observations in various cell lines (e.g., U2OS cells) that showed the majority of DRP1 puncta were associated with mitochondria [26,27]. Importantly, a similar analysis using RyR2 (Figure 1D) revealed that ~71% of DRP1 puncta were also located within 198 nm from RyR2, suggesting a strong proximity to jSR domains, suggesting that a significant number of DRP1 oligomers are accumulated at the proximity of the jSR.

We next used pre-embedding immunogold transmission electron microscopy (TEM) labeling of DRP1 (DRP1–IG) to reveal its subcellular localization at higher resolution. This allowed us to investigate whether the mitochondrial DRP1 pool is enriched specifically at mitochondria–jSR–T-tubule contact sites (Ca^2+^ release units). For an easier view, the key organelles are pseudo-colored: orange for the SR, blue for the T-tubule, and green for the mitochondria. As highlighted by the zoomed area with red arrows pointing at DRP1–IG particles, the larger-size particles appeared to be located more abundantly in the area where mitochondria, jSR, and T-tubule were in proximity (Figure 1E). These DRP1 IG-positive particles are specific in labeling DRP1 as they were significantly diminished in the no-primary antibody control and DRP1icKO samples (Figure S2D). For a better understanding of the quantitative analysis of the location of DRP1–IG particles at the different regions of mitochondria, a schematic diagram illustrates the T side (transversal side) of mitochondria, where jSR, T-tubule, and mitochondria are in juxtaposition, and L side (longitudinal side) of mitochondria, where jSR is not in proximity (Figure 1F and Figure S4). As shown in Figure 1E,F, DRP1 immunogold particles were predominantly located on the transversal (T) side of mitochondria—where jSR and T-tubules are adjacent—compared to the longitudinal (L) side, which lacks jSR contacts. Quantification revealed that although T sides represent only ~1/3 of the mitochondrial perimeter (Figure 1H), they contained 74 ± 4.05% of all DRP1 particles (Figure 1G). After normalization, DRP1 density at T sides was 8.09 ± 1.68-fold higher than L sides (Figure 1I), confirming the significant enrichment at Ca^2+^ release units. Altogether, these data show that the majority of mitochondria-bound DRP1 is localized at the mitochondria–jSR contacts, in proximity to the Ca^2+^ release units [29]. The smaller proportion of mitochondria-bound DRP1 on the L side is likely localized at the mitochondria–nSR contacts, as will be shown next.

### 3.2. Mitochondria–SR Contacts Harbor the Membrane-Bound DRP1

**Figure 2:**
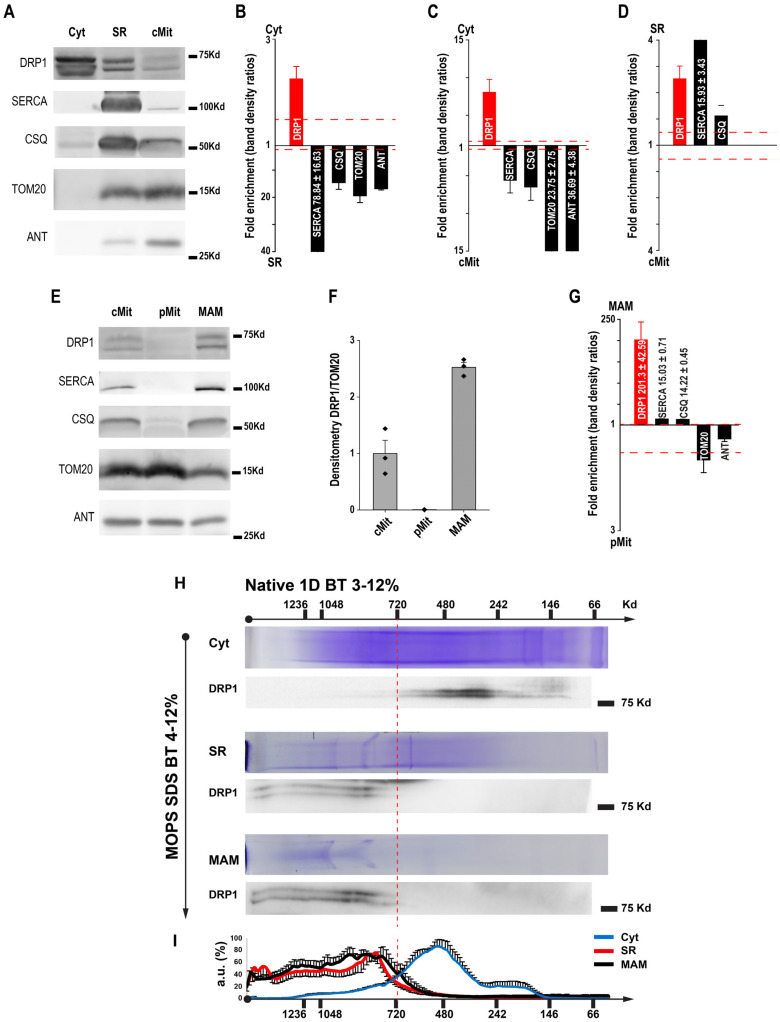
DRP1 is localized at the SR and MAM fractions. (**A**) Representative Western blot of the cardiac subcellular fractions obtained by differential centrifugation, cytosol (Cyt), sarcoplasmic reticulum (SR), and crude mitochondria (cMit). The sarcoplasmic/endoplasmic proteins reticulum ATPase (SERCA) and calsequestrin (CSQ) were used as SR markers, while mitochondrial import receptor subunit (TOM20) and adenine nucleotide translocase (ANT) were used as outer and inner mitochondrial membrane markers, respectively (OMM and IMM). (**B**–**D**) Comparisons of relative abundance of proteins between the Cyt, SR, and cMit fractions. (**E**) Representative Western blot of the mitochondrial fractions obtained by Percoll^®^ purification. The same protein markers as in panel A were used. (**F**) Western blot analysis of the mitochondrial fractions showed a DRP1 enrichment in the mitochondria–SR associations (MAM) but not in the pure mitochondrial fraction (pMit). (**G**) Comparison of relative abundance of proteins between the pMit and MAM fractions. N = 3 fractionations. (**H**) Solubilized proteins from the cytosol, MAM, and SR subcellular fractions were separated according to their masses on a linear 3–12% acrylamide gradient gel for BN-PAGE (first dimension, 1D). Native protein complexes were separated by 4–12% Bis-Tris SDS-PAGE gradient gel (second dimension, 2D). (**I**) Densitometry analysis of the DRP1 oligomers distribution in the different fractions. N = 3–5 fractionations.

The observation that DRP1 preferentially localizes at the mitochondria–jSR contacts prompted us to further determine the DRP1 presence in subcellular fractions (isolated by differential centrifugation and Percoll gradient purification, Figure S1) of heart ventricle homogenates by Western blot. The results indicate that DRP1 is differently distributed among the cytosol (Cyt), SR, and crude mitochondria (cMit) fractions (Figure 2A). The Cyt fraction was validated by the absence of SERCA (sarco-endoplasmic reticulum Ca^2+^ ATPase), CSQ (calsequestrin), TOM20, and ANT (adenine nucleotide translocator); the SR fraction was validated by the highest abundance of SERCA and CSQ and the lower abundance of TOM20 and ANT than in the cMit fraction; the cMit fraction was validated by the highest abundance of TOM20 and ANT and the lower abundance of SERCA and CSQ than in the SR fraction. These characteristics of each cardiac subcellular fraction are consistent with our previously reported subfractionations [37]. Note that the cardiac SR is a structurally diverse organelle that consists of junctional (RyR2 and CSQ-enriched) and network (SERCA-enriched) SR (jSR and nSR, respectively) [43]. Unfortunately, no biochemical method can perfectly separate jSR and nSR; therefore, the SR subfraction in this study was a mixture of both components. Furthermore, it has been extensively reported that sarcoplasmic reticulum–endoplasmic reticulum (SR–ER) and mitochondria are tightly tethered by several proteins [44,45,46] and that SR/ER can “wrap” around the mitochondria [47], resulting in cross-“contamination” between the SR and mitochondrial subfraction. Accordingly, the SR fraction contains mitochondrial proteins and vice versa. Based on these principles, quantitative comparisons of the relative amount of DRP1, SERCA, CSQ, TOM20, and ANT, between the Cyt versus the SR and cMit fractions were plotted (Figure 2B,C). The analysis showed that DRP1 was 2.25 ± 0.23-fold higher in the cytosol than in SR (Figure 2B) and 8.05 ± 1.72-fold higher in the Cyt than in cMit (Figure 2C). Surprisingly, when comparing the SR versus cMit fraction, DRP1 was 3.64 ± 0.26-fold higher in the SR than in the cMit fraction (Figure 2D), suggesting a significant accumulation of membrane-bound DRP1 in the SR fraction. The presence of DRP1 in the SR fraction could result from the presence of mitochondrial membrane in the SR fraction and/or inherent existence. However, the accumulation of DRP1 in the SR fraction is a peculiarity of the heart. When we compared SR and ER fractions from the heart and liver, respectively, no traces of DRP1 and VDAC were detected in the liver (Figure S5). Similarly, no mitochondrial VDAC and cytochrome C were found in the ER fraction of MEF [48], supporting the idea of DRP1-specific participation in the SR–mitochondria contacts in the heart.

The cMit fraction was then further purified using a Percoll^®^ gradient separating pMit from MAM (Figure 2E) to elucidate the DRP1 positioning within these two sub-mitochondrial regions. DRP1 was undetectable in the pMit fraction; the SR markers SERCA and CSQ were also undetectable. In contrast, most of DRP1 from the cMit was recovered in the MAM fraction, which also contains both SR (SERCA and CSQ) and mitochondrial markers (TOM20 and ANT) (Figure 2E). The normalized densitometry of DRP1 indicated a high presence of DRP1 in the MAM, with no detectable traces of DRP1 in the pMit (2.52 ± 0.08 vs. 0.13 ± 0.035, respectively, Figure 2F). The relative fold enrichments of DRP1, SERCA, CSQ, TOM20, and ANT between MAM and pMit are plotted in Figure 2G, showing that DRP1 is highly enriched in the MAM versus the pMit fraction (201.3 ± 42.6-fold enrichment). These results indicate that, in cardiac tissue, mitochondria-bound DRP1 exclusively localizes at the MAM.

### 3.3. DRP1 Localized at the SR and MAM Fractions at High Oligomeric State

As described before, the main difference between the soluble and membrane-bound DRP1 resides in its oligomerization state [49]. Therefore, a comprehensive analysis to elucidate the oligomerization states of the DRP1 in the different cellular subfractions was performed. We first solubilized DRP1 complexes from the different fractions using digitonin and resolved by Blue Native (BN)-PAGE. Solubilized proteins from the Cyt, SR, and MAM fractions were separated according to their size on a linear 3–12% BN-PAGE gradient gel (first dimension, 1stD) (Figure 2H, Coomassie Blue lanes). Native protein complexes were then separated by 4–12% Bis-Tris SDS-PAGE gradient gel (second dimension, 2ndD) (Figure 2H, blots). The densitometry analysis of the 2D experiments revealed that the SR and MAM fractions contained higher molecular weight DRP1 complexes, in comparison with the smaller DRP1 complexes found in the Cyt fraction (Figure 2I). No DRP1 was detected in the 2ndD gel from pMit (Figure S6). The non-overlapping oligomeric nature of the DRP1 in the soluble fraction versus the membranous fractions confirms that large DRP1 oligomers attach to membranous fractions, while small DRP1 oligomers remain soluble in the ytosol. Additionally, the experiment ruled out the possibility of cross-contamination between the cytosol and membranous fractions.

Overall, these data, together with the IF and DRP1–IG results, indicate that highly oligomerized DRP1 is preferentially localized at the SR and MAM but not in the cytosol or in the bulk mitochondrial surface represented by the pMit fraction.

### 3.4. Ca^2+^ Transient Activity Is Involved in DRP1 Positioning at the Mitochondria–SR Contacts in the Heart

**Figure 3:**
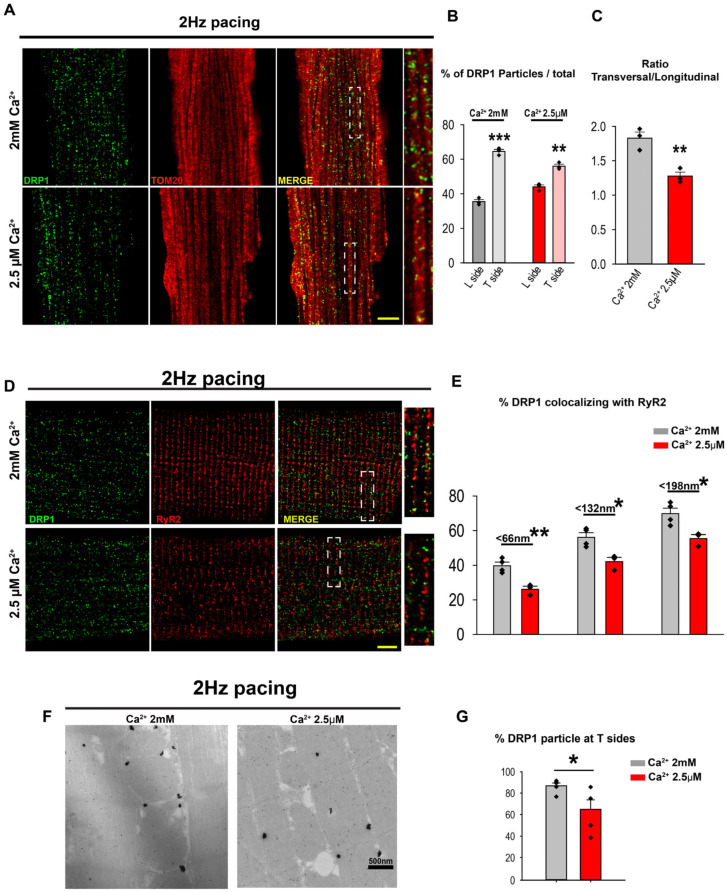
Ca^2+^ transients take part in DRP1 positioning at the cardiac mitochondria–SR contacts. Cardiomyocytes were isolated and submitted to electrical field stimulation (15 min–2 Hz) while being superfused Ca^2+^ (2 mM) or quasi-Ca^2+^-free (2.5 μM) buffer. (**A**) Zoomed images (scale bar = 5µm) are after AiryScan processing DRP1 (green) and Tom20 (red). (**B**,**C**) Analysis of DRP1 puntae distribution along the mitochondrial transversal (T) and longitudinal (L) sides. (N = 3 animals; 10 cells per animal) *: 0.05 > *p* > 0.01, **: 0.01 ≥ *p* ≥ 0.001, ***: p < 0.001. (**D**) Zoomed images (scale bar = 5 µm) are after AiryScan processing DRP1 (green) and RyR2 (red). (**E**) Summarized data showing the fraction of DRP1 within the indicated distance ranges (<66, <132, and <198 nm) from RyR2. (N = 3 animals; 10 cells per animal) *: 0.05 > *p* > 0.01, **: 0.01 ≥ *p* ≥ 0.001. (**F**) Immunogold–DRP1particles in field-stimulated ACM perfused with different [Ca^2+^]. (**G**) Analysis of the IG–DRP1 particle distributions found at the mitochondrial T sides and sub-mitochondrial fractions of beating rat heart perfused with either 1.8 mM Ca^2+^ buffer or quasi-Ca^2+^ free (2.5 µM) for 60 min. Two-tailed Student’s t-test was used for comparisons between two independent sample groups.

It has been reported that mitochondrial Ca^2+^ signaling contributes significantly to the positioning of other Ca^2+^-related proteins, such as the MCUC [37]. Therefore, the high Ca^2+^ microdomains created at the mitochondria–SR contacts could be a key factor implicated in the DRP1 accumulation at SR and MAM fractions. To test this idea, ACM were isolated and acutely submitted to 15 min–2 Hz electrical field stimulation while being continuously perfused with Ca^2+^ (2 mM) or quasi-Ca^2+^-free (2.5 μM) extracellular buffer. Cells were immediately fixed and immunolabeled for DRP1 and TOM20 (Figure 3A) or RyR2 (Figure 3D). In the IF images, the distribution of DRP1 particles aggregated at the L and T sides of the mitochondria (TOM20, red) was analyzed. The results showed a significantly higher amount of DRP1 oligomers at the T sides of the mitochondria when ACM were stimulated in the presence of 2 mM Ca^2+^ in comparison with those stimulated with 2.5 μM Ca^2+^ (Figure 3B,C). Similarly, the analysis of DRP1 distribution along the RyR2 from isolated ACM revealed a significant amount of DRP1 particles at the RyR2 vicinity upon field stimulation in the presence of 2 mM Ca^2+^ (Figure 3E). In the original binarized mask (66 nm), there was 39.71 ± 2.00% of DRP1 colocalized with RyR2 in the presence of 2 mM Ca^2+^. This colocalization decreased to 26.08 ± 1.75% in the condition of 2.5 µM Ca^2+^, a 1.58-fold difference. In the area within 1 pixel distance (132 nm), the colocalization was 56.11 ± 2.67% in 2 mM Ca^2+^ and 41.97 ± 2.48% in 2.5 µM Ca^2+^, a 1.38-fold difference. This difference became even smaller at 192 nm (1.29-fold difference). This progressive decrease in the differences of percentage of DRP1 and RyR2 colocalization as their separation distance becomes larger suggests that Ca^2+^-mediated DRP1 accumulation in the vicinity of RyR2 requires mitochondria–SR in close contact, where the microdomains of Ca^2+^ concentrations are high.

To further validate Ca^2+^-dependent DRP1 accumulation in the mitochondria–SR contacts, we performed IG-TEM analysis from field-stimulated ACM (Figure 3F). The obtained data showed a significant reduction of the DRP1 particles at the T side of the mitochondria in ACM perfused with a 2.5 µM Ca^2+^ in comparison with those perfused with 2 mM Ca^2+^ (86.84 ± 2.72% vs. 64.83 ± 8.82%, Figure 3G).

**Figure 4:**
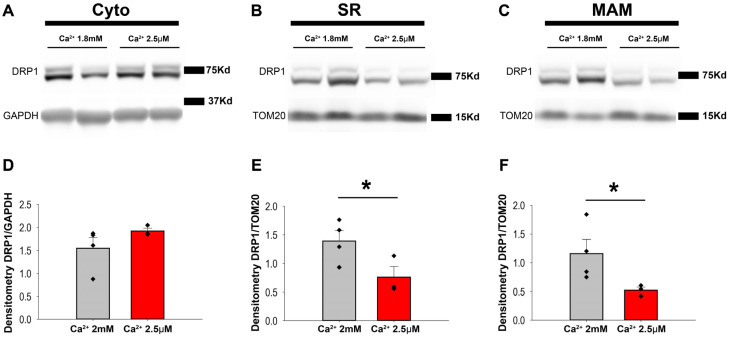
Ca^2+^ transients alter the DRP1 distribution along the fractions. Representative Western blot and quantification of DRP1 levels in the cytosol (**A**,**D**), SR (**B**,**E**), and MAM (**C**,**F**). GAPDH and TOM20 were used as loading controls for the cytosolic and organellar fractions, respectively. N = 3–4 rats per condition. *: 0.05 > *p* > 0.01. One-tailed Student’s t-test was used for comparisons between two independent sample groups.

To confirm that data obtained in isolated ACM were also applicable to the whole heart preparations, spontaneously beating rat hearts were Langendorff-perfused with Krebs buffer supplemented with 2.5 µM or 1.8 mM Ca^2+^ for 60 min before being subjected to homogenization and fractionation. Western blot analysis of the fractions showed that cytosolic DRP1 levels were similar under two different Ca^2+^ concentrations in the perfusion buffer (Figure 4A–C). Consistent with the previous results, the levels of DRP1 were significantly lower in the SR and MAM fractions from the hearts treated with low Ca^2+^ buffer (1.39 ± 0.36 vs. 0.76 ± 0.18 for the SR and 1.16 ± 0.28 vs. 0.52 ± 0.05 for the MAM, Figure 4D–F). In addition, no DRP1 in the pMit was detected under both Ca^2+^ concentration treatment (Figure S7).

Taken together, these results suggest that the high [Ca^2+^] at the dyads stabilizes high molecular weight DRP1 complexes positioned within the mitochondria–SR contacts in the beating heart, preventing its delocalization.

### 3.5. β-ACTIN Is Responsible for DRP1 Accumulation in MAM

**Figure 5:**
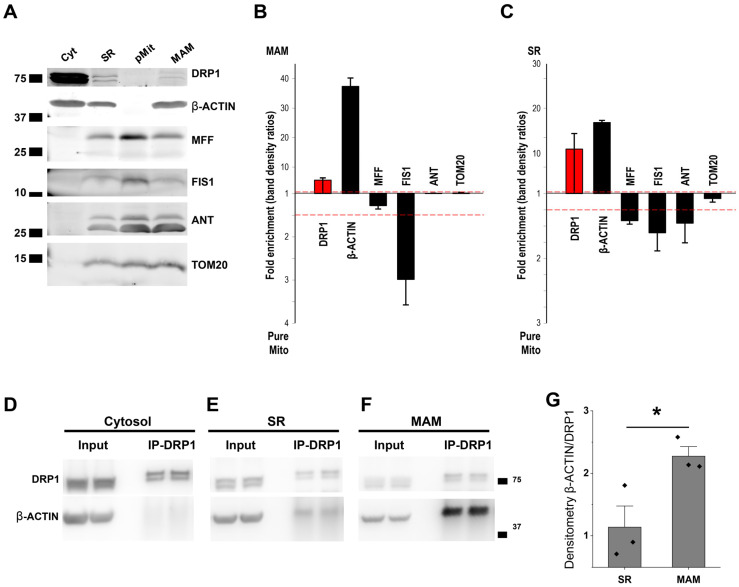
β-ACTIN follows DRP1 distribution among the cellular fractions and co-precipitates with DRP1 exclusively at the mitochondria–SR contacts. (**A**) Representative WB of DRP1-anchoring proteins—MFF and FIS1—distribution among the different cellular fractions and (**B**,**C**) comparisons of relative abundance (Cyt, SR, pMit, and MAM, N = 5 animals per fractionation, 3 different fractionations). (**D**–**F**) DRP1-IP and β-ACTIN Co-IP for Cyt, SR, and MAM fractions. (**G**) WB quantification of β-ACTIN coimmunoprecipitating with DRP1 in the SR and MAM fractions (N = 5 animals per fractionation, 3 different fractionations for each DRP1-IP, *: 0.05 > *p* > 0.01). Two-tailed Student’s t-test was used for comparisons between two independent sample groups.

Besides Ca^2+^, DRP1 positioning at the mitochondrial membrane strictly depends on anchoring proteins [50,51,52,53,54]. We studied the distribution among the fractions of the previously described DRP1 anchoring proteins, such as mitochondrial fission factor (MFF), mitochondrial fission 1 (FIS1), (Figure 5A,C), and mitochondrial dynamic proteins 49 and 51 (MiD49 and MiD51) (Figure S8). Surprisingly, Western blot analysis showed that, in the adult murine heart, none of the previously proposed anchoring proteins were enriched on MAM and SR fractions or followed DRP1 distribution among the subcellular fractions. Although these proteins may be involved in recruiting DRP1 in cardiac mitochondria, it is likely that other proteins related to Ca^2+^ signaling may anchor DRP1 at MAM.

It has been shown that β-ACTIN polymerization at or near fission sites can stimulate oligomeric maturation of DRP1 on mitochondria filaments and thus may serve as a dynamic reservoir for recruiting oligomerized DRP1 to the mitochondria–ER contacts in U2OS cells [55]. In addition, increased intracellular Ca^2+^ concentrations induce β-ACTIN polymerization [56].

**Figure 6:**
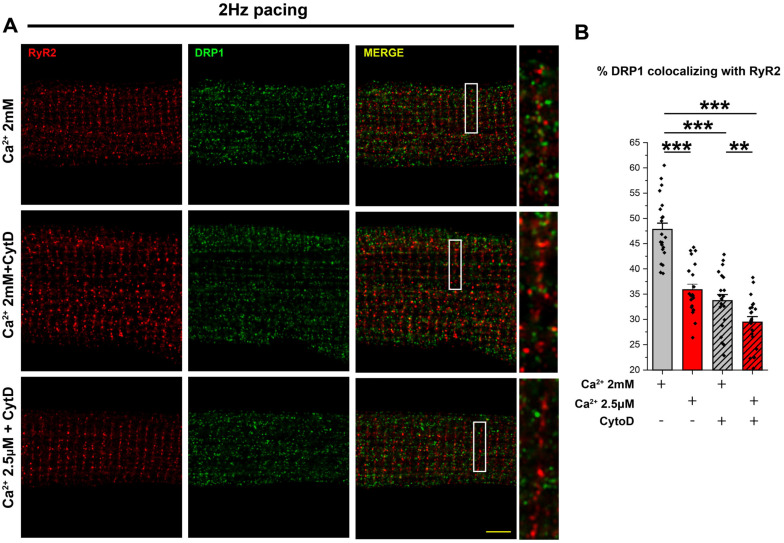
Disruption of β-ACTIN filaments misplaces the DRP1 localization. (**A**) Cardiomyocytes were isolated and pretreated with cytochalasin D 10 μM for 30 min in stand and culture medium. Then the cells were submitted to electrical field stimulation (30 min–1 Hz) while being superfused Ca^2+^ (2 mM) or quasi-Ca^2+^-free (2.5 μM) buffer in the presence or absence of cytochalasin D 10μM. Zoomed images (scale bar = 5 µm) and detail are after AiryScan processing DRP1 (green) and RyR2 (red). (**B**) Summarized data showing the fraction of DRP1 within the indicated distance ranges (<66 nm) from RyR2. (N = 3 animals; 10 cells per animal) **: 0.01 ≥ *p* ≥ 0.001, ***: *p* < 0.001. Factorial-nested ANOVA analysis was applied for comparison by a priori comparisons.

Therefore, we determined whether DRP1 and β-ACTIN interact directly, the spatial specificity of their interactions at the MAM, and the regulatory role of Ca^2+^ in this process in the heart. By performing DRP1 immunoprecipitation from cytosolic, SR, and MAM fractions, we observed that, despite the high abundance of DRP1 and β-ACTIN in the cytosol (Figure 5C), β-ACTIN was only co-immunoprecipitated with DRP1 in the membranous fractions, SR, and MAM (Figure 5D–G). Interestingly, a significantly higher specific interaction between DRP1 and β-ACTIN was present in the MAM when compared with the SR fraction (Figure 5F–G), suggesting a significantly stronger binding between DRP1 and β-ACTIN at the MAM. Lastly, we studied the relevance of DRP1–β-ACTIN interaction in ACM. To confirm this strong interaction, we use cytochalasin D, a toxin-compound known to bind ACTIN filaments inhibiting both the association and dissociation of subunits. By treating freshly isolated ACM with cytochalasin D under pacing and continuously perfused with 2 mM or 2.5 µM Ca^2+^, we determined DRP1 positioning at the RyR2 vicinity. In the presence of cytochalasin D, DRP1 was delocalized from the RyR2 vicinity when ACM were perfused with 2mM Ca^2+^, and this delocalization was exacerbated when ACM were perfused with 2.5 µM Ca^2+^ (Figure 6A,B). These data support the hypothesis that β-ACTIN and Ca^2+^ are interdependently responsible for the DRP1 accumulation in MAM.

### 3.6. MAM-Localized DRP1 and β-ACTIN Are Sustained in DRP1-Deficient Hearts

Finally, to test whether DRP1 positioning at mitochondria–SR contact sites was dependent on total protein abundance, we analyzed DRP1icKO hearts 6 weeks after tamoxifen-induced deletion. Surprisingly, we found that although DRP1 expression was markedly reduced in total tissue and cytosol, DRP1 was better preserved in the MAM fraction, suggesting selective retention or stabilization of DRP1 at this site.

**Figure 7:**
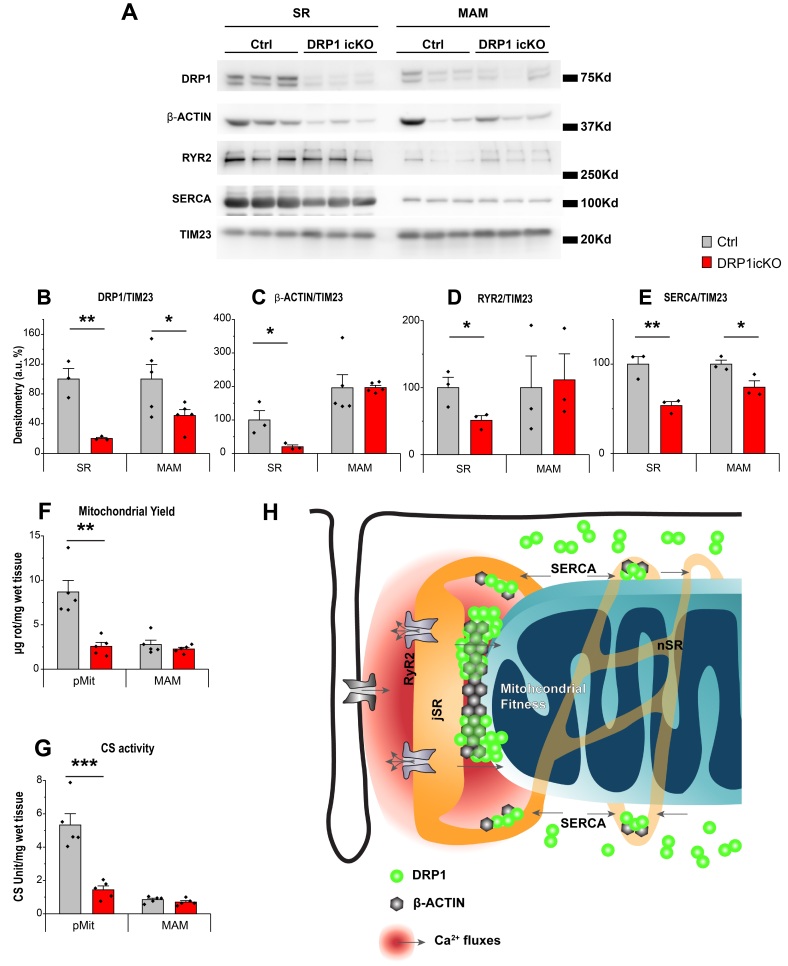
DRP1 levels are better preserved at the MAM fraction in the DRP1icKO mice. (**A**) Western blot analysis of SR and MAM fractions obtained from control and DRP1icKO animals after 6 weeks of tamoxifen injection. (**B**–**E**) Quantification of DRP1, β-ACTIN, RyR2, and SERCA levels in the SR and MAM fractions from both genotypes (N = 5 animals per fractionation, 3 different fractionations per genotype, *: 0.05 > *p* > 0.01, **: 0.01 ≥ *p* ≥ 0.001). (**F**) Mitochondrial yield and (**G**) citrate synthase activity with respect to wet tissue from pMit and MAM obtained from control and DRP1icKO animals after 6 weeks of tamoxifen injection (N = 5 animals per fractionation, 5 different fractionations per genotype, **: 0.01 ≥ *p* ≥ 0.001, ***: *p* < 0.001). (**H**) Mechanisms of high molecular weight DRP1 clusters at the SR and MAM in the beating heart. Schematic representation of the proposed mechanism by which Ca^2+^.

As mentioned before, the DRP1 protein levels decreased 84.8% in DRP1icKO heart tissue after 6 weeks of tamoxifen injection when compared with control animals (Figures S1 and S9A). Despite this significant DRP1 depletion, our DRP1icKO mice were able to survive for 12 weeks, comparable to those reported previously [16,35]. Western blot analysis of DRP1 levels in different cellular fractions from DRP1icKO hearts shows only minimal traces of DRP1 in the cytosolic fraction (8.7% respect to the Ctrl, Figure S9B). At the SR fraction, a decrease of 79.9% was observed in the DRP1icKO animals. This diminished DRP1 expression was concomitantly accompanied by a significant decrease of β-ACTIN of the same magnitude (Figure 7A–C). Interestingly, the decreases of DRP1 and β-ACTIN in the MAM fraction were much smaller (49%) in the DRP1icKO animals, as if DRP1 tried to “hang in there” and be “the last one to leave” under the adverse conditions of DRP1 deletion. Because of the changes in Ca^2+^ transients in cardiomyocytes from DRP1icKO, we also determined the expression levels of the main proteins involved in the SR Ca^2+^ handling: RyR2 and SERCA. Following the tendencies of DRP1 and β-ACTIN, RyR2 and SERCA showed significantly reduced expression in the SR fraction from the DRP1icKO (~50% for both proteins). In the MAM fraction, RyR2 levels were maintained while the SERCA levels showed a milder decrease when compared with the SR fraction changes (Figure 7D,E). We also observed that the mitochondrial pool and citrate synthase (CS) activity relative to the cardiac tissue (Figure 7F,G) was dramatically decreased in the pMit fraction (72.2% less in the DRP1icKO vs. Ctrl) while it appeared unaltered in the MAM fractions, where the DRP1 levels are better preserved.

These data, together with the IP results, suggest that the strong interaction between β-ACTIN and DRP1 in the MAM helps to preserve its positioning at this fraction, even in the face of DRP1 ablation. The unique feature of DRP1 oligomers positioning at the high Ca^2+^ functional microdomains between jSR and mitochondria, in strong association with β-ACTIN, could enable DRP1 to exert its non-canonical role in regulating cellular energetics, Ca^2+^ signaling, and contractility and, as such, preserves cardiac function for sustaining the lifespan of these animals.

## 4. Discussion

This study demonstrates for the first time that, under physiological conditions in both ACM and ex vivo perfused hearts, DRP1 forms high molecular weight oligomers, localizes specifically at mitochondria–jSR contacts (MAM), and is absent from SR-free pure mitochondria (pMit). This distinct localization pattern is driven by the strong interactions between DRP1 and β-ACTIN as well as the modulation by Ca^2+^ signals. Previously, we have reported the strategic localization of mitochondrial protein complexes involved in [Ca^2+^]_m_ dynamics and ECB coupling, which either clustered (MCU) to or excluded (NCLX) from the mitochondria–jSR contacts [37,40]. Here, we report that DRP1 oligomers accumulate in this exquisite area through interacting with β-ACTIN and the regulation of EC coupling-associated Ca^2+^ transients. The structural and functional microdomains between mitochondria and jSR are the hubs not only for a privileged Ca^2+^ exchange [29] but also for other key cellular activities, such as redox signaling, lipid transfer, and mitochondria quality control [57,58,59,60,61]. The clustering of high molecular weight DRP1 protein complexes in this narrow space between these two organelles may enable them to locally regulate these crucial cellular activities and help to preserve mitochondrial fitness under stress conditions [62].

DRP1 levels in the SR fraction are ~3 folds higher than that in cMit (Figure 2). Our data also show that SR fraction contains mostly the SR membrane, with a smaller amount of tethered mitochondrial membranes based on the quantitation of each organelle’s resident proteins (Figure 2A,D), a phenomenon typically observed in the cardiac tissue (Figure S5). Therefore, the cardiac SR appears to contain a significant amount of DRP1. The existence of DRP1 in ER has been observed previously. An earlier study suggested that ER-localized DRP1 participates in the formation of nascent secretory vesicles from the ER cisternae [63]. In line with this observation, recent studies in cell lines have reported that some of the DRP1 anchoring proteins are found not only in the mitochondria but also at the ER surface [17,27,64]. In U2OS cells, ER can function as a platform for DRP1 oligomerization and transferring to mitochondria for mitochondrial fission [17,26,27]. Finally, a recent report showed that DRP1 directly shapes peripheral ER tubules to facilitate ER–mitochondria interactions to promote mitochondrial division [65]. Future experiments are needed to determine the functional roles of SR-associated DRP1 in the heart. As described before by others, the main difference between the soluble and membrane-bound DRP1 rests on its oligomerization state [49,66]. In addition to high-resolution imaging and Western blot measurements to show the unique DRP1 distribution at the jSR–mitochondria contacts, our results from Blue Native Page gels further demonstrate that in the heart, higher DRP1 complexes are found in the SR and MAM fractions. In comparison, significantly smaller complexes are present in the Cyt fraction. Therefore, these experiments rule out the possibility that the membranous SR and MAM fractions were contaminated with the cytosolic form of DRP1 (Figure 2H,I).

The detailed mechanism responsible for the preferential accumulation of DRP1 at the mitochondria–jSR contacts still remains elusive. Our biochemical analyses, based on relative enrichments, indicate that both the SR and MAM fractions contain a mixture of junctional and network SR components. However, the MAM fraction shows a relative enrichment in jSR markers—specifically, RyR2 and the L-type Ca^2+^ channel α1.2—compared to the network SR marker SERCA (Figure S10). This study’s findings suggest that the high Ca^2+^ concentrations in the jSR–mitochondria microdomains may facilitate DRP1 accumulation. Indeed, Ca^2+^ transients from the beating heart are crucial for the DRP1 positioning (Figure 3 and Figure 4).

Besides Ca^2+^ transients as part of the DRP1 positioning mechanism, we studied the distribution of the most relevant DRP1 anchoring proteins to elucidate whether they follow the same pattern as DRP1, acting as a landing platform to recruit DRP1 to mitochondria–SR contacts. It has been previously described that DRP1 assembles to the outer mitochondrial membrane in a GTP-binding, hydrolysis, and nucleotide-exchanging-dependent manner [50] and by interacting with adaptors and anchoring proteins such as MFF, FIS1, and MiD49/MiD51 [51,52,53,54]. Our Western blot results from subcellular fractions demonstrate that most of these previously described anchoring proteins appear to be enriched in the pMit fraction, where DRP1 is undetectable (Figure 5). Therefore, these anchoring proteins might be implicated in docking functions; however, they are not defining the final positioning of DRP1 at the mitochondria–SR contacts in ACM. We also observed that only β-ACTIN has similar distribution as DRP1 among the cellular fractions (Figure 5). We have demonstrated that the distribution of both proteins is due to a strong and direct interaction between DRP1 and β-ACTIN at the MAM fraction (Figure 5). These data support the important role of β-ACTIN in docking DRP1 in MAM. It has been shown that β-ACTIN oligomerization enhances ER–mitochondria interaction, allowing a more efficient Ca^2+^ transport from ER to mitochondria through MCU [19]. Also, Ca^2+^ is an important regulator for β-ACTIN polymerization [67]. Our results showed that Ca^2+^ dependent DRP1 co-localization with RyR2 is reduced by β-ACTIN depolymerization with cytochalasin D (Figure 6), indicating that Ca^2+^ transients-mediated DRP1 positioning at the jSR–mitochondria contacts is partially mediated by Ca^2+^-dependent β-ACTIN polymerization. Recent reports propose that, in cell culture models, β-ACTIN and its associated proteins, like INF2 and SPIRE1C, play a pivotal role in DRP1 recruitment to the nucleation sites in ER and mitochondria, respectively, with Ca^2+^ playing a critical role in the process [17,18,19,26,27]. Whether these proteins play any roles in DRP1 accumulation in the MAM of ACM remains unclear since their distribution among the cellular fractions is different from DRP1 and β-ACTIN.

Finally, we have observed that even under conditions of severe DRP1 depletion (as in DRP1icKO mice), DRP1 and β-ACTIN remain selectively preserved in the MAM fraction, alongside key Ca^2+^-handling proteins such as RyR2 and SERCA. This strategic retention suggests a hierarchized localization of DRP1, with mitochondria–SR junctions serving as prioritized zones critical for sustaining mitochondrial signaling and energetics. The observed preservation of mitochondrial yield and CS activity at MAMs, in contrast to their loss in pure mitochondrial fractions, supports this functional prioritization. Since the DRP1 anchor mechanism to the MAMs is still unknown and will require future investigation, it is hard to predict how DRP1 misslocalization would affect the heart physiology either under basal conditions or under stress. A few studies report the heart phenotype upon DRP1 ablation [16,68,69,70], but none of them address the consequences of DRP1 miss-positioning. One would expect changes in the mitochondrial physiology or performance under stressful conditions that would require higher energy demand. However, as mentioned, future investigation is required to manipulate the DRP1 preferential location at the heart.

In summary, this study describes the novel findings that, in ACM, membrane-bound DRP1 forms very high molecular weight complexes in association with β-ACTIN and is strategically localized at jSR–mitochondria contacts. During excitation–contraction cycles, Ca^2+^ transients promote DRP1 oligomerization and accumulation at the mitochondria–SR contacts. Interestingly, this process appears to lead to modest fission events. In the context of marked DRP1 deficiency, our findings indicate that DRP1 localization at MAM is not passively dictated by total abundance but rather reflects active recruitment and stabilization, potentially through interaction with β-ACTIN and local Ca^2+^ signaling. This mechanism may help maintain mitochondrial–SR communication and energetics, even under conditions of DRP1 depletion (Figure 7H).

This study also raises several questions for future investigation. In particular, what specific proteins in the DRP1-associated high molecular weight complex (DRP1–β-ACTIN interactome) are critical for its structural arrangement in the ACM? Which post-translational modifications are involved in [Ca^2+^]_c_-dependent DRP1 strategic positioning? Importantly, what are the molecular mechanisms of the DRP1-β-ACTIN complex in regulating excitation–bioenergetics coupling and Ca^2+^ communication between SR and mitochondria? As mentioned above, it is still unknown whether the DRP1–β-ACTIN interaction is direct or mediated by undefined additional proteins. It is certain that mitochondrial-bound components are required to keep DRP1 at the jSR–mitochondria interface. To further investigate possible interactors and regulatory roles of the DRP1 complexome, higher resolution gels to resolve the interactome would be needed (large pore blue native PAGE (LP-BN-PAGE) and complexome profiling [71]).

As final conclusions, our findings reveal that DRP1 localization in adult cardiomyocytes is spatially regulated by an actin-based mechanism in coordination with calcium signaling during excitation–contraction (E–C) coupling cycles. This dual regulation facilitates the recruitment of DRP1 to mitochondria–junctional SR contact sites, ensuring proper mitochondrial positioning in the context of dynamic cardiac activity. By identifying this E–C coupling-linked mechanism of DRP1 anchoring, our study provides new insight into how mitochondrial-associated DRP1 is spatially and functionally integrated into cardiomyocyte physiology.

The specific localization of DRP1 at mitochondria–sarcoplasmic reticulum (SR) contact sites raises important questions about the functional relevance of this subcellular positioning. The mitochondria-associated membrane (MAM) region is increasingly recognized as a critical hub for calcium signaling and metabolic regulation [39,72,73,74]. Therefore, the preferential localizations of highly oligomerized DRP1 in this high Ca^2+^ microdomain could interact with nearby proteins to exert key signaling mechanisms. Notably, our previous work demonstrated that DRP1 regulates cardiac bioenergetics through modulation of the transient openings of mitochondrial permeability transition pore (mPTP), shown to be triggered by high Ca^2+^ and ROS [25]. This raises the intriguing possibility that DRP1 localization at MAMs is functionally linked to its role in metabolic regulation. Future studies should directly test this hypothesis. Indeed, investigations to dissect the causal relationship between DRP1 subcellular localization and its role in cardiac bioenergetics are currently underway in our laboratory.

## Data Availability

The data presented in this study are available on request from the corresponding author.

## Supplementary Materials

The following supporting information can be downloaded at: https://www.mdpi.com/article/10.3390/cells14161259/s1, Figure S1. Scheme showing the different components of the cardiac subcellular fractions obtained by differential centrifugation and Percoll gradient purification. Figure S2. A. Verification of DRP1 antibody and DRP1icKO mice in IF and WB from freshly isolated cardiomyocytes lysate. Anti-DRP1 antibody (BD Biosciences, 611112) detects significalntly less mitochondrial associated punctate DRP1 “hot spots” DRP1cKO cardiomyocytes after 6 weeks tamoxifen induction (left panel). On the right, WB analysis from cardiomyocytes lysate from control and tamoxifen induced mice, following the same trend. B. Verification of DRP1 antibody in MEF cells. Anti-DRP1 antibody (BD Biosciences, 611112) detects mitochondrial associated punctate DRP1 “hot spots” in wild type but not DRP1 KO MEFs. Note that in DRP1 KO cells, mitochondria are dramatically elongated. C. Verification of DRP1 antibody (BD Biosciences, 611112) in freshly isolated cardiomyocytes from rat treatd with DRP1shRNA. D. Verification of DRP1 IG secondary antibody signal. Figure S3. Confocal images of 4× zoom cardiomyocytes regions (scale bar 0.5 mm) showing the mitochondrial pattern (TOM20, red, upper left panel) and DRP1 pattern (green, middle left panel). Selected mitochondria for study are labeled in yellow (upper panel, right) and overlapped with the DRP1 mask (5% max intensity threshold). In the lower panels, in higher magnification, the spatial resolution and how each mitochondrion was divided into 5 different regions. The DRP1 thresholded particles touching the mitochondria OMM at the three areas in the middle were considered at the longitudinal side, while the ones touching the mitochondria OMM at the 2 areas in the mitochondrial poles were considered at the transversal side. Figure S4. Electron microscopy micrograph of left ventricular papillary muscle showing cardiac interfibrillar mitochondria (green), T-tubules (blue) junctional sarcoplasmic reticulum at the transversal side (orange) of the mitochondria, and network sarcoplasmic reticulum marking the longitudinal side (pink) of the mitochondria. Figure S5. Comparison of DRP1 distribution among SR fractions from hear and Liver. Note that the mitochondria (VDAC) are not detected in the Liver ER fraction in comparison with the high abundance of mitochondria in this fraction in the heart. Figure S6. Solubilized proteins from the cytosol, MAM and SR subcellular fractions were separated according to their masses on a linear 3–12% acrylamide gradient gel for BN-PAGE (first dimension, 1D). Native protein complexes were separated by 4–12% Bis-Tris SDS-PAGE gradient gel (second dimension, 2D). Figure S7. In rat hearts perfused with 2.5 μM Ca^2+^ or 1.8 mM Ca^2+^ the DRP1 decrease in SR and MAM fractions was not related with DRP1 presence in the pMito fraction. Figure S8. Representative WB of DRP1 proposed anchoring proteins–β-ACTIN, MiD49 and MiD51-showing distribution among the different cellular fractions (Cyt, SR, pMit and MAM, N = 5 animals per fractionation, 3 different fractionations) SERCA, HSP60 and DRP1 were used as quality control of the fracitionation process. Figure S9. WB analysis and quantification of DRP1 levels of the control and Drp1icko animals in (A) Total homogenate and (B) Cytosolic fraction (each number corresponds to a single mice, N = 5 per group). Figure S10. (A) Representative Western blot of total cardiac homogenate (TH) and cardiac cellular fractions obtained by differential centrifugation, cytosol (Cyt), sarcoplasmic reticulum (SR), crude mitochondria (cMit) pure mitochondria (pMit) and mitochondria-associated membranes (MAM). RyR2 and L-type Ca^2+^ channels were used as junctional SR markers while SERCA was used as network SR marker. (B,C) Comparison of relative abundance of junctional SR and network SR proteins between SR and MAM fractions. N = 3 fractionations from 5 mice hearts each.

## References

[1] Friedman J.R., Nunnari J. (2014). Mitochondrial Form and Function. Nature.

[2] Kasahara A., Scorrano L. (2014). Mitochondria: From Cell Death Executioners to Regulators of Cell Differentiation. Trends Cell Biol..

[3] Pham A.H., Meng S., Chu Q.N., Chan D.C. (2012). Loss of Mfn2 Results in Progressive, Retrograde Degeneration of Dopaminergic Neurons in the Nigrostriatal Circuit. Hum. Mol. Genet..

[4] Lee S., Sterky F.H., Mourier A., Terzioglu M., Cullheim S., Olson L., Larsson N.-G. (2012). Mitofusin 2 Is Necessary for Striatal Axonal Projections of Midbrain Dopamine Neurons. Hum. Mol. Genet..

[5] Wasiak S., Zunino R., McBride H.M. (2007). Bax/Bak Promote Sumoylation of DRP1 and Its Stable Association with Mitochondria during Apoptotic Cell Death. J. Cell Biol..

[6] Rambold A.S., Kostelecky B., Elia N., Lippincott-Schwartz J. (2011). Tubular Network Formation Protects Mitochondria from Autophagosomal Degradation during Nutrient Starvation. Proc. Natl. Acad. Sci. USA.

[7] Burté F., Carelli V., Chinnery P.F., Yu-Wai-Man P. (2015). Disturbed Mitochondrial Dynamics and Neurodegenerative Disorders. Nat. Rev. Neurol..

[8] Wang W., Karamanlidis G., Tian R. (2016). Novel Targets for Mitochondrial Medicine. Sci. Transl. Med..

[9] Dorn II G.W. (2015). Mitochondrial Dynamism and Heart Disease: Changing Shape and Shaping Change. EMBO Mol. Med..

[10] Yoon Y., Pitts K.R., McNiven M.A. (2001). Mammalian Dynamin-like Protein DLP1 Tubulates Membranes. Mol. Biol. Cell.

[11] Mishra P., Chan D.C. (2016). Metabolic Regulation of Mitochondrial Dynamics. J. Cell Biol..

[12] Basu K., Lajoie D., Aumentado-Armstrong T., Chen J., Koning R.I., Bossy B., Bostina M., Sik A., Bossy-Wetzel E., Rouiller I. (2017). Molecular Mechanism of DRP1 Assembly Studied in Vitro by Cryo-Electron Microscopy. PLoS ONE.

[13] Imoto M., Tachibana I., Urrutia R. (1998). Identification and Functional Characterization of a Novel Human Protein Highly Related to the Yeast Dynamin-like GTPase Vps1p. J. Cell. Sci..

[14] Santel A., Frank S., Gaume B., Herrler M., Youle R.J., Fuller M.T. (2003). Mitofusin-1 Protein Is a Generally Expressed Mediator of Mitochondrial Fusion in Mammalian Cells. J. Cell. Sci..

[15] Dorn G.W. (2019). Evolving Concepts of Mitochondrial Dynamics. Annu. Rev. Physiol..

[16] Song M., Mihara K., Chen Y., Scorrano L., Dorn G.W. (2015). Mitochondrial Fission and Fusion Factors Reciprocally Orchestrate Mitophagic Culling in Mouse Hearts and Cultured Fibroblasts. Cell Metab..

[17] Korobova F., Ramabhadran V., Higgs H.N. (2013). An Actin-Dependent Step in Mitochondrial Fission Mediated by the ER-Associated Formin INF2. Science.

[18] Manor U., Bartholomew S., Golani G., Christenson E., Kozlov M., Higgs H., Spudich J., Lippincott-Schwartz J. (2015). A Mitochondria-Anchored Isoform of the Actin-Nucleating Spire Protein Regulates Mitochondrial Division. eLife Sci..

[19] Chakrabarti R., Ji W.-K., Stan R.V., de Juan Sanz J., Ryan T.A., Higgs H.N. (2018). INF2-Mediated Actin Polymerization at the ER Stimulates Mitochondrial Calcium Uptake, Inner Membrane Constriction, and Division. J. Cell Biol..

[20] Song M., Dorn G.W. (2015). Mitoconfusion: Noncanonical Functioning of Dynamism Factors in Static Mitochondria of the Heart. Cell Metab..

[21] Ishihara T., Ban-Ishihara R., Maeda M., Matsunaga Y., Ichimura A., Kyogoku S., Aoki H., Katada S., Nakada K., Nomura M. (2015). Dynamics of Mitochondrial DNA Nucleoids Regulated by Mitochondrial Fission Is Essential for Maintenance of Homogeneously Active Mitochondria during Neonatal Heart Development. Mol. Cell. Biol..

[22] Papanicolaou K.N., Kikuchi R., Ngoh G.A., Coughlan K.A., Dominguez I., Stanley W.C., Walsh K. (2012). Mitofusins 1 and 2 Are Essential for Postnatal Metabolic Remodeling in Heart. Circ. Res..

[23] Silva Ramos E., Larsson N.-G., Mourier A. (2016). Bioenergetic Roles of Mitochondrial Fusion. Biochim. Biophys. Acta.

[24] Shirakabe A., Zhai P., Ikeda Y., Saito T., Maejima Y., Hsu C.-P., Nomura M., Egashira K., Levine B., Sadoshima J. (2016). Drp1-Dependent Mitochondrial Autophagy Plays a Protective Role Against Pressure Overload-Induced Mitochondrial Dysfunction and Heart Failure. Circulation.

[25] Zhang H., Wang P., Bisetto S., Yoon Y., Chen Q., Sheu S.-S., Wang W. (2017). A Novel Fission-Independent Role of Dynamin-Related Protein 1 in Cardiac Mitochondrial Respiration. Cardiovasc. Res..

[26] Ji W., Hatch A.L., Merrill R.A., Strack S., Higgs H.N. (2015). Actin Filaments Target the Oligomeric Maturation of the Dynamin GTPase Drp1 to Mitochondrial Fission Sites. eLife.

[27] Ji W.-K., Chakrabarti R., Fan X., Schoenfeld L., Strack S., Higgs H.N. (2017). Receptor-Mediated Drp1 Oligomerization on Endoplasmic Reticulum. J. Cell Biol..

[28] Dorn G.W. (2013). Mitochondrial Dynamics in Heart Disease. Biochim. Biophys. Acta.

[29] Sharma V.K., Ramesh V., Franzini-Armstrong C., Sheu S.S. (2000). Transport of Ca^2+^ from Sarcoplasmic Reticulum to Mitochondria in Rat Ventricular Myocytes. J. Bioenerg. Biomembr..

[30] Glancy B., Balaban R.S. (2012). Role of Mitochondrial Ca^2+^ in the Regulation of Cellular Energetics. Biochemistry.

[31] Luongo T.S., Lambert J.P., Yuan A., Zhang X., Gross P., Song J., Shanmughapriya S., Gao E., Jain M., Houser S.R. (2015). The Mitochondrial Calcium Uniporter Matches Energetic Supply with Cardiac Workload during Stress and Modulates Permeability Transition. Cell Rep..

[32] Rasmussen T.P., Wu Y., Joiner M.A., Koval O.M., Wilson N.R., Luczak E.D., Wang Q., Chen B., Gao Z., Zhu Z. (2015). Inhibition of MCU Forces Extramitochondrial Adaptations Governing Physiological and Pathological Stress Responses in Heart. Proc. Natl. Acad. Sci. USA.

[33] Wang P., Fernandez-Sanz C., Wang W., Sheu S.-S. (2018). Why Don’t Mice Lacking the Mitochondrial Ca^2+^ Uniporter Experience an Energy Crisis?. J. Physiol..

[34] Hom J., Yu T., Yoon Y., Porter G., Sheu S.-S. (2010). Regulation of Mitochondrial Fission by Intracellular Ca^2+^ in Rat Ventricular Myocytes. Biochim. Biophys. Acta.

[35] Ikeda Y., Shirakabe A., Maejima Y., Zhai P., Sciarretta S., Toli J., Nomura M., Mihara K., Egashira K., Ohishi M. (2015). Endogenous Drp1 Mediates Mitochondrial Autophagy and Protects the Heart against Energy Stress. Circ. Res..

[36] Ishihara N., Nomura M., Jofuku A., Kato H., Suzuki S.O., Masuda K., Otera H., Nakanishi Y., Nonaka I., Goto Y.-I. (2009). Mitochondrial Fission Factor Drp1 Is Essential for Embryonic Development and Synapse Formation in Mice. Nat. Cell Biol..

[37] De La Fuente S., Fernandez-Sanz C., Vail C., Agra E.J., Holmstrom K., Sun J., Mishra J., Williams D., Finkel T., Murphy E. (2016). Strategic Positioning and Biased Activity of the Mitochondrial Calcium Uniporter in Cardiac Muscle. J. Biol. Chem..

[38] O’Connell T.D., Rodrigo M.C., Simpson P.C. (2007). Isolation and Culture of Adult Mouse Cardiac Myocytes. Methods Mol. Biol..

[39] Fernandez-Sanz C., Ruiz-Meana M., Miro-Casas E., Nuñez E., Castellano J., Loureiro M., Barba I., Poncelas M., Rodriguez-Sinovas A., Vázquez J. (2014). Defective Sarcoplasmic Reticulum-Mitochondria Calcium Exchange in Aged Mouse Myocardium. Cell Death Dis..

[40] De La Fuente S., Lambert J.P., Nichtova Z., Fernandez Sanz C., Elrod J.W., Sheu S.-S., Csordás G. (2018). Spatial Separation of Mitochondrial Calcium Uptake and Extrusion for Energy-Efficient Mitochondrial Calcium Signaling in the Heart. Cell Rep..

[41] Sánchez J.A. (2013). Role of Connexin 43 in Ischemia-Reperfusion Injury.

[42] Matsuoka Y., Srere P.A. (1973). Kinetic Studies of Citrate Synthase from Rat Kidney and Rat Brain. J. Biol. Chem..

[43] Segretain D., Rambourg A., Clermont Y. (1981). Three Dimensional Arrangement of Mitochondria and Endoplasmic Reticulum in the Heart Muscle Fiber of the Rat. Anat. Rec..

[44] Chen Y., Csordás G., Jowdy C., Schneider T.G., Csordás N., Wang W., Liu Y., Kohlhaas M., Meiser M., Bergem S. (2012). Mitofusin 2-Containing Mitochondrial-Reticular Microdomains Direct Rapid Cardiomyocyte Bioenergetic Responses via Interorganelle Ca^2+^ Crosstalk. Circ. Res..

[45] Dorn G.W. (2020). Mitofusins as Mitochondrial Anchors and Tethers. J. Mol. Cell Cardiol..

[46] Naon D., Scorrano L. (2014). At the Right Distance: ER-Mitochondria Juxtaposition in Cell Life and Death. Biochim. Biophys. Acta.

[47] Friedman J.R., Lackner L.L., West M., DiBenedetto J.R., Nunnari J., Voeltz G.K. (2011). ER Tubules Mark Sites of Mitochondrial Division. Science.

[48] Wieckowski M.R., Giorgi C., Lebiedzinska M., Duszynski J., Pinton P. (2009). Isolation of Mitochondria-Associated Membranes and Mitochondria from Animal Tissues and Cells. Nat. Protoc..

[49] Macdonald P.J., Stepanyants N., Mehrotra N., Mears J.A., Qi X., Sesaki H., Ramachandran R. (2014). A Dimeric Equilibrium Intermediate Nucleates Drp1 Reassembly on Mitochondrial Membranes for Fission. Mol. Biol. Cell.

[50] Kalia R., Wang R.Y.-R., Yusuf A., Thomas P.V., Agard D.A., Shaw J.M., Frost A. (2018). Structural Basis of Mitochondrial Receptor Binding and Constriction by DRP1. Nature.

[51] Fekkes P., Shepard K.A., Yaffe M.P. (2000). Gag3p, an Outer Membrane Protein Required for Fission of Mitochondrial Tubules. J. Cell Biol..

[52] Mozdy A.D., McCaffery J.M., Shaw J.M. (2000). Dnm1p GTPase-Mediated Mitochondrial Fission Is a Multi-Step Process Requiring the Novel Integral Membrane Component Fis1p. J. Cell Biol..

[53] Tieu Q., Nunnari J. (2000). Mdv1p Is a WD Repeat Protein That Interacts with the Dynamin-Related GTPase, Dnm1p, to Trigger Mitochondrial Division. J. Cell Biol..

[54] van der Bliek A.M. (2000). A Mitochondrial Division Apparatus Takes Shape. J. Cell Biol..

[55] Hatch A.L., Ji W.-K., Merrill R.A., Strack S., Higgs H.N. (2016). Actin Filaments as Dynamic Reservoirs for Drp1 Recruitment. Mol. Biol. Cell.

[56] Wales P., Schuberth C.E., Aufschnaiter R., Fels J., García-Aguilar I., Janning A., Dlugos C.P., Schäfer-Herte M., Klingner C., Wälte M. (2016). Calcium-Mediated Actin Reset (CaAR) Mediates Acute Cell Adaptations. eLife.

[57] Davidson S.M., Duchen M.R. (2006). Calcium Microdomains and Oxidative Stress. Cell Calcium.

[58] Ruiz-Meana M., Fernandez-Sanz C., Garcia-Dorado D. (2010). The SR-Mitochondria Interaction: A New Player in Cardiac Pathophysiology. Cardiovasc. Res..

[59] Kaludercic N., Deshwal S., Di Lisa F. (2014). Reactive Oxygen Species and Redox Compartmentalization. Front. Physiol..

[60] Vance J.E. (2015). Phospholipid Synthesis and Transport in Mammalian Cells. Traffic.

[61] Boyman L., Karbowski M., Lederer W.J. (2020). Regulation of Mitochondrial ATP Production: Ca^2+^ Signaling and Quality Control. Trends Mol. Med..

[62] Wang W., Fernandez-Sanz C., Sheu S.-S. (2018). Regulation of Mitochondrial Bioenergetics by the Non-Canonical Roles of Mitochondrial Dynamics Proteins in the Heart. Biochim. Biophys. Acta Mol. Basis Dis..

[63] Yoon Y., Pitts K.R., Dahan S., McNiven M.A. (1998). A Novel Dynamin-like Protein Associates with Cytoplasmic Vesicles and Tubules of the Endoplasmic Reticulum in Mammalian Cells. J. Cell Biol..

[64] Prudent J., McBride H.M. (2016). Mitochondrial Dynamics: ER Actin Tightens the Drp1 Noose. Curr. Biol..

[65] Adachi Y., Kato T., Yamada T., Murata D., Arai K., Stahelin R.V., Chan D.C., Iijima M., Sesaki H. (2020). Drp1 Tubulates the ER in a GTPase-Independent Manner. Mol. Cell.

[66] Francy C.A., Clinton R.W., Fröhlich C., Murphy C., Mears J.A. (2017). Cryo-EM Studies of Drp1 Reveal Cardiolipin Interactions That Activate the Helical Oligomer. Sci. Rep..

[67] Takeshita N., Evangelinos M., Zhou L., Serizawa T., Somera-Fajardo R.A., Lu L., Takaya N., Nienhaus G.U., Fischer R. (2017). Pulses of Ca^2+^ Coordinate Actin Assembly and Exocytosis for Stepwise Cell Extension. Proc. Natl. Acad. Sci. USA.

[68] Song M., Franco A., Fleischer J.A., Zhang L., Dorn G.W. (2017). Abrogating Mitochondrial Dynamics in Mouse Hearts Accelerates Mitochondrial Senescence. Cell Metab..

[69] Piao L., Fang Y.-H., Fisher M., Hamanaka R.B., Ousta A., Wu R., Mutlu G.M., Garcia A.J., Archer S.L., Sharp W.W. (2024). Dynamin-Related Protein 1 Is a Critical Regulator of Mitochondrial Calcium Homeostasis during Myocardial Ischemia/Reperfusion Injury. FASEB J..

[70] Wu D., Hu Q., Li H., Yin Y., Wang P., Wang W. (2025). Drp1 Knockdown Aggravates Obesity-Induced Cardiac Dysfunction and Remodeling. Mitochondrion.

[71] Wittig I., Malacarne P.F. (2021). Complexome Profiling: Assembly and Remodeling of Protein Complexes. Int. J. Mol. Sci..

[72] Kohlhaas M., Maack C. (2013). Calcium Release Microdomains and Mitochondria. Cardiovasc. Res..

[73] Vance J.E. (2014). MAM (Mitochondria-Associated Membranes) in Mammalian Cells: Lipids and Beyond. Biochim. Biophys. Acta.

[74] Maack C., O’Rourke B. (2007). Excitation-Contraction Coupling and Mitochondrial Energetics. Basic Res. Cardiol..

