# Supplementary material for: Highly Oligomeric DRP1 Strategic Positioning at Mitochondria–Sarcoplasmic Reticulum Contacts in Adult Murine Heart Through ACTIN Anchoring
Source: Cells. 2025 Aug 14;14(16):1259. doi: 10.3390/cells14161259 (PMC12384166; doi:10.3390/cells14161259)
Supplement: Supplementary file 1 [file cells-14-01259-s001.zip › cells-3724917-supplementary.pdf]

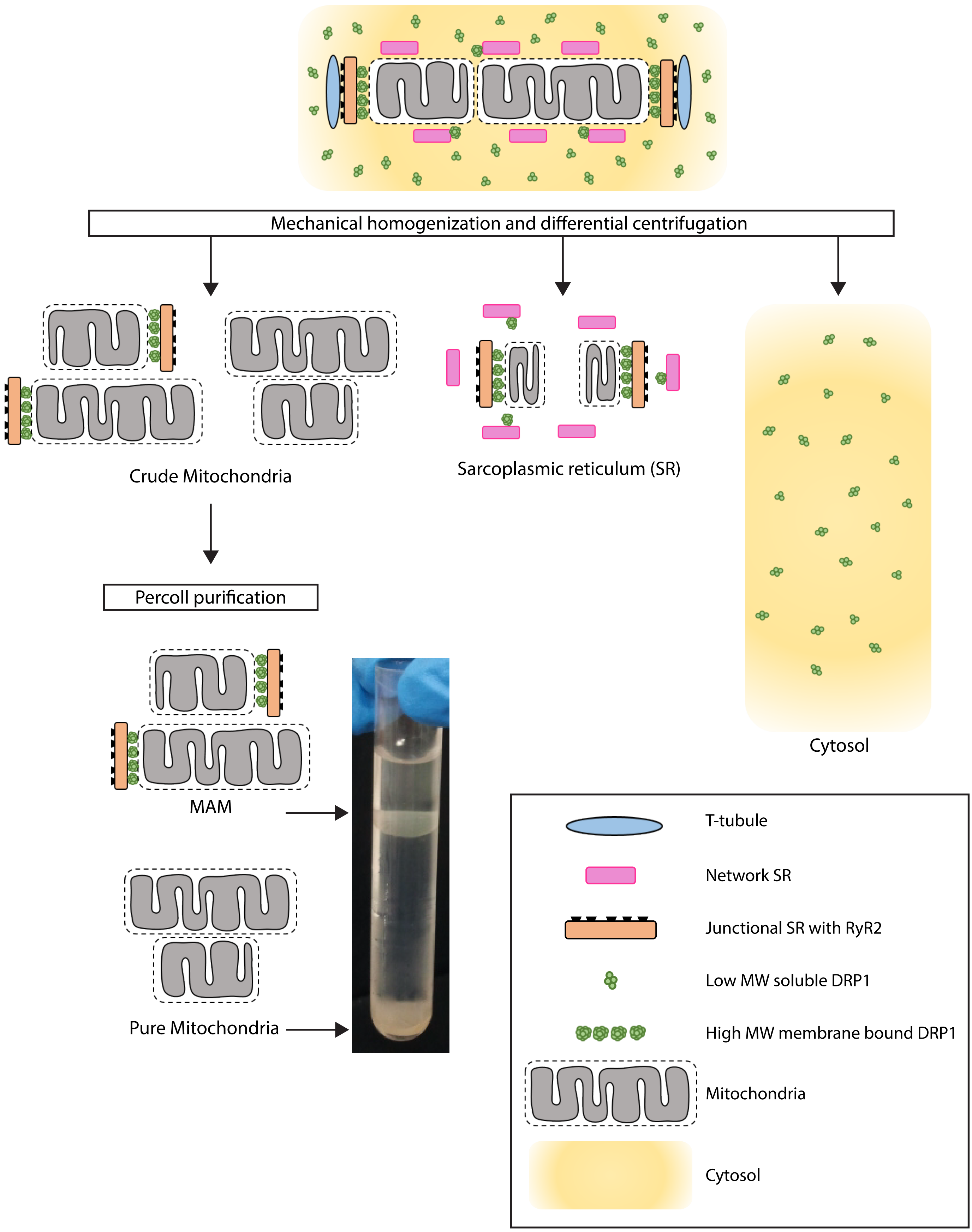

Figure S1. Scheme showing the different components of the cardiac subcellular fractions obtained by differential centrifuga-tion and Percoll gradient purification.

Supplementary Figure S2

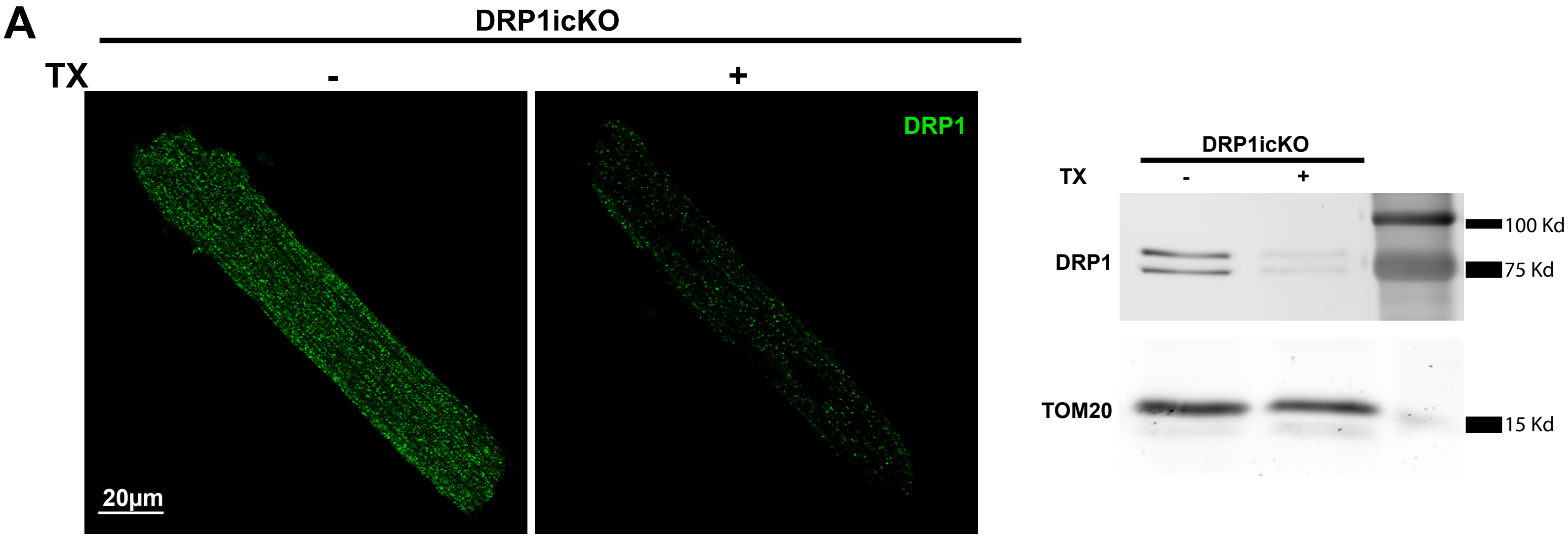

Figure S2A. Verification of DRP1 antibody and DRP1icKO mice in IF and WB from freshly isolated cardiomyocytes lysate. Anti-DRP1 antibody (BD Biosciences, 611112) detects significalntly less mitochondrial associated punctate DRP1 "hot spots" DRP1cKO cardiomyocytes after 6 weeks tamoxifen induction (left panel). On the right, WB analysis from cardiomyocytes lysate from control and tamoxifen induced mice, following the same trend.

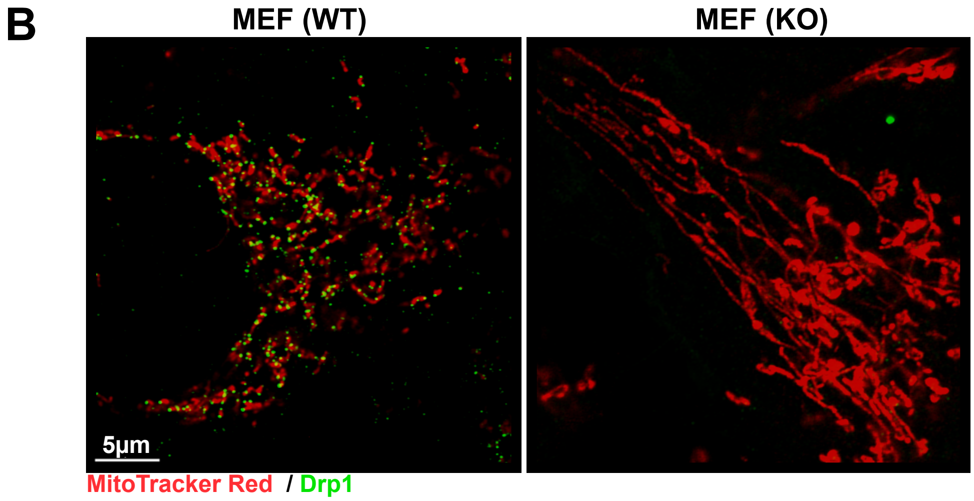

Figure S2B. Verification of DRP1 antibody in MEF cells. Anti-DRP1 antibody (BD Biosciences, 611112) detects mitochondrial associated punctate DRP1 "hot spots" in wild type but not DRP1 KO MEFs. Note that in DRP1 KO cells, mitochondria are dramatically elongated.

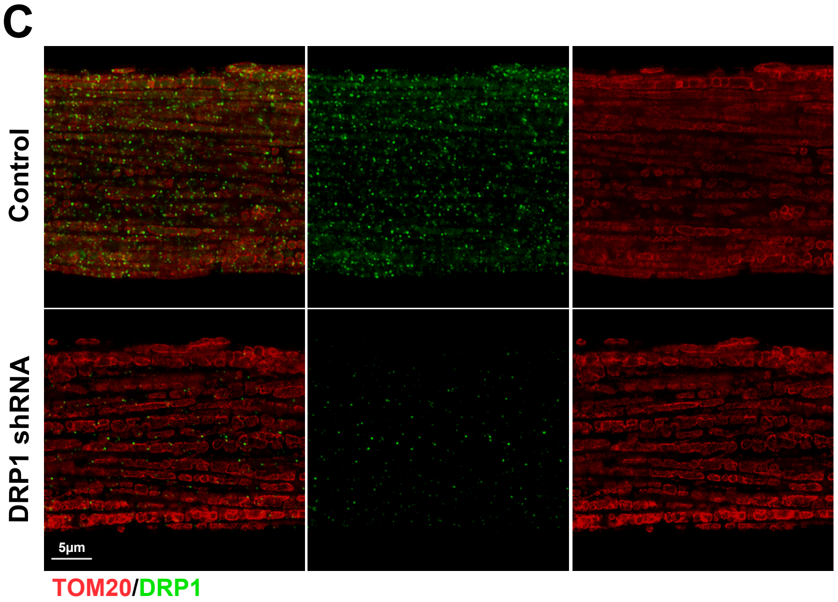

Figure S2C. Verification of DRP1 antibody (BD Biosciences, 611112) in freshly isolated cardiomyocytes from rat treatd with DRP1shRNA

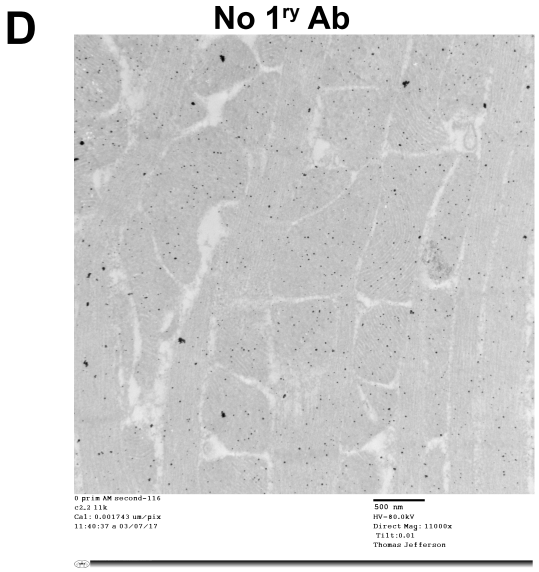

Figure S2D. Verification of DRP1 IG secondary antibody signal.

Supplementary Figure S3

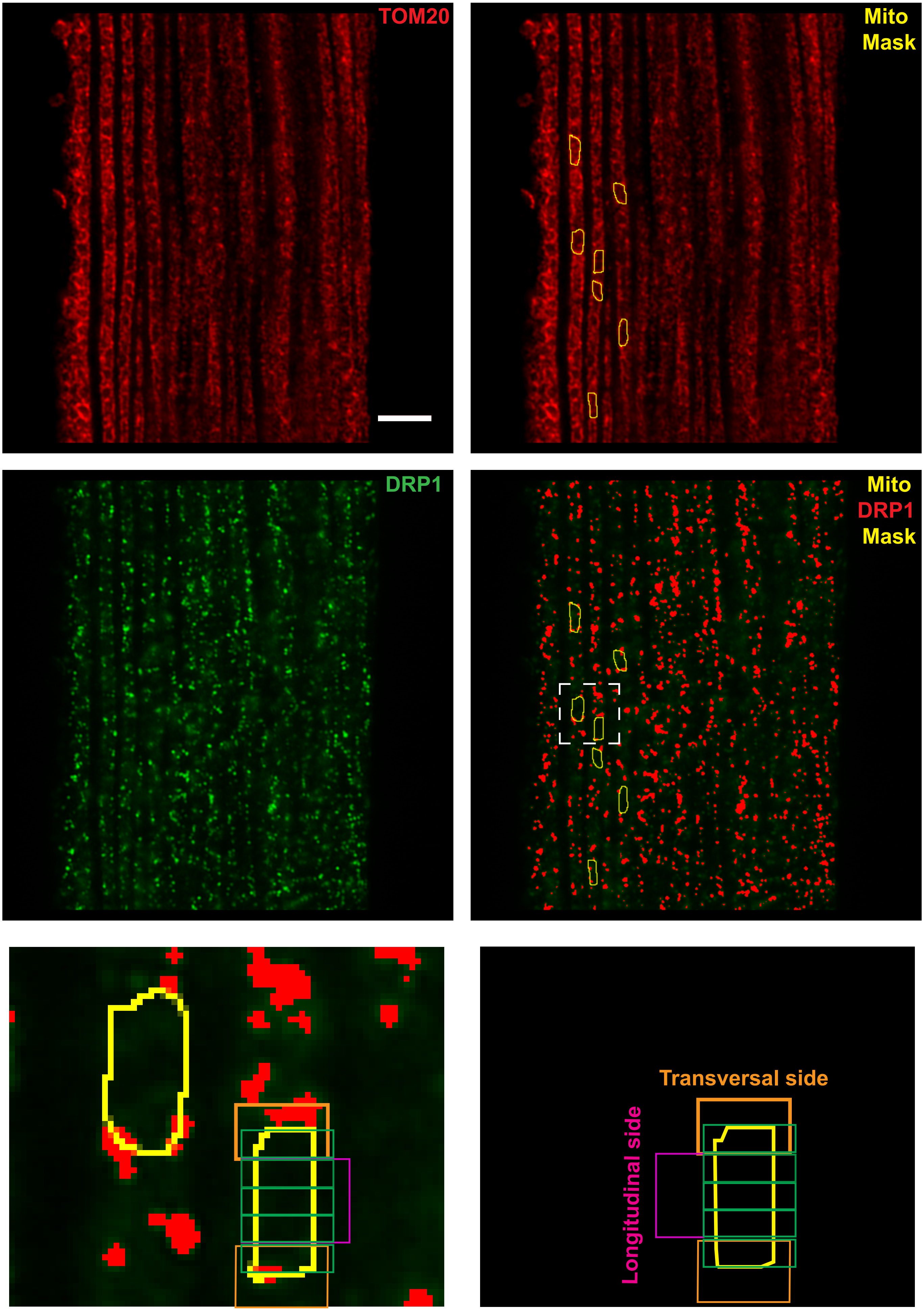

Figure S3. Confocal images of 4x zoom cardiomyocytes regions (scale bar 0.5mm) showing the mitochondrial pattern (TOM20, red, upper left panel) and DRP1 pattern (green, middle left panel). Selected mitochondria for study are labeled in yellow (upper panel, right) and overlapped with the DRP1 mask (5% max intensity threshold). In the lower panels, in higher magnification, the spatial resolution and how each mitochondrion was divided into 5 different regions. The DRP1 thresholded particles touching the mitochondria OMM at the three areas in the middle were considered at the longitudinal side, while the ones touching the mitochondria OMM at the 2 areas in the mitochondri-al poles were considered at the transversal side.

Supplementary Figure S4

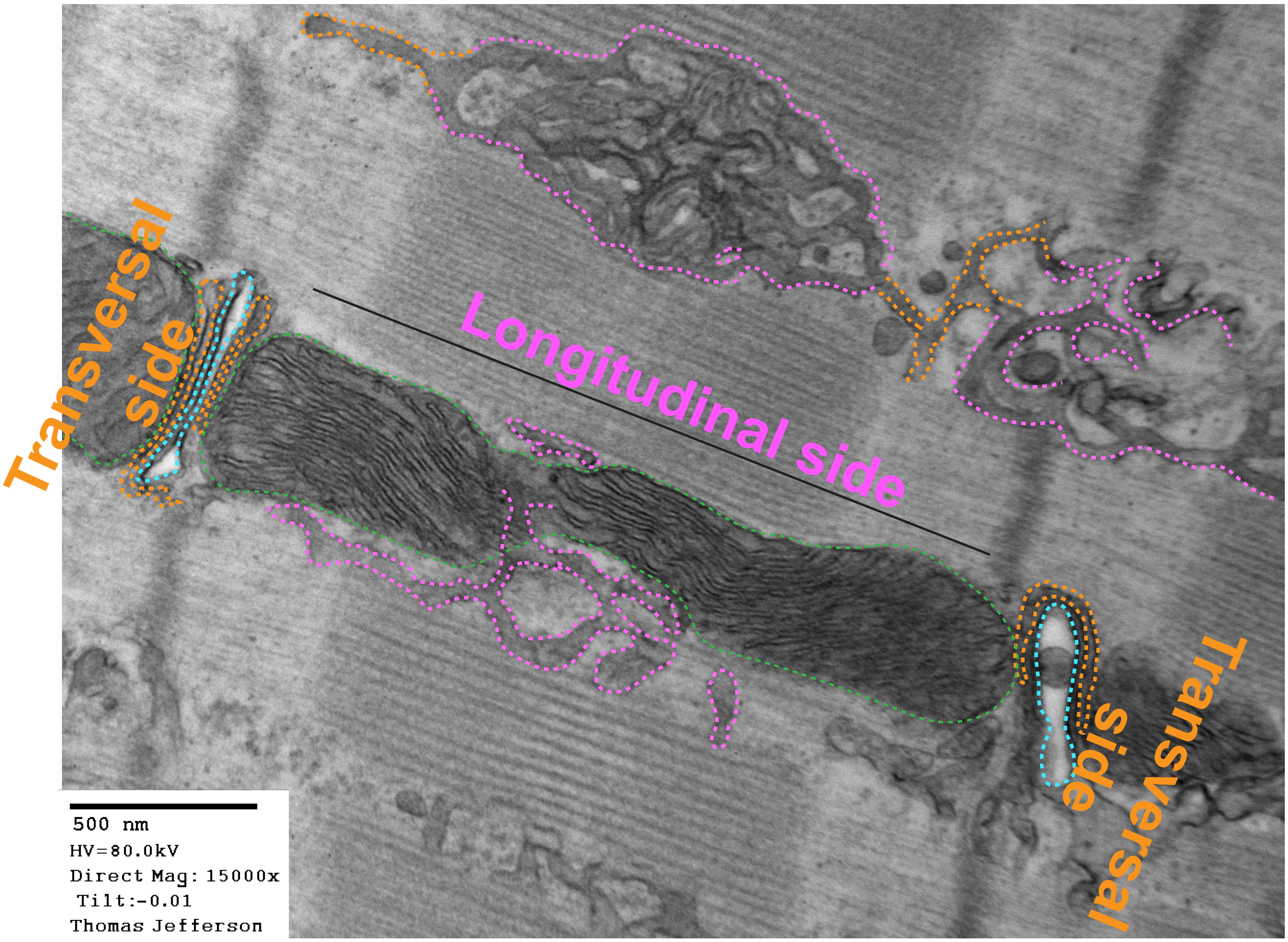

Figure S4. Electron microscopy micrograph of left ventricular papillary muscle showing cardiac interfibrillar mitochon-dria (green), T-tubules (blue) junctional sarcoplasmic reticulum at the transversal side (orange) of the mitochondria ,and network sarcoplasmic reticulum marking the longitudinal side (pink) of the mitochondria.

Supplementary Figure S5

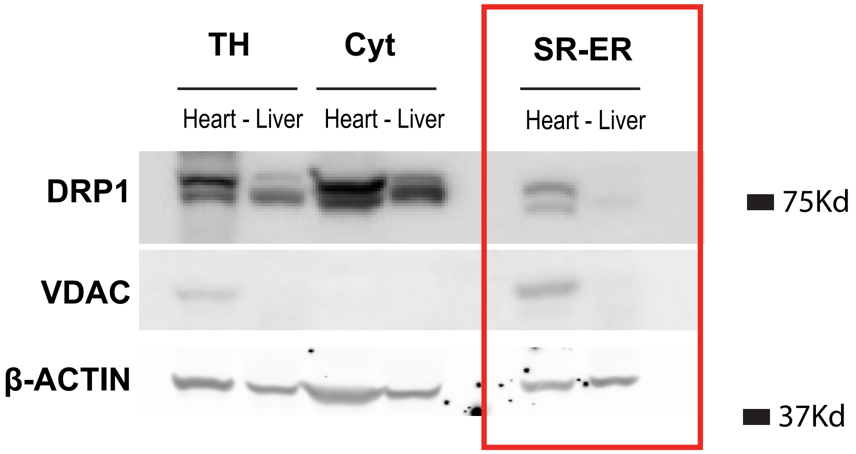

Figure S5. Comparison of DRP1 distribution among SR fractions from hear and Liver. Note that the mitochondria (VDAC) are not detected in the Liver ER fraction in comparison with the high abundance of mitochondria in this fraction in the heart.

Supplementary Figure S6

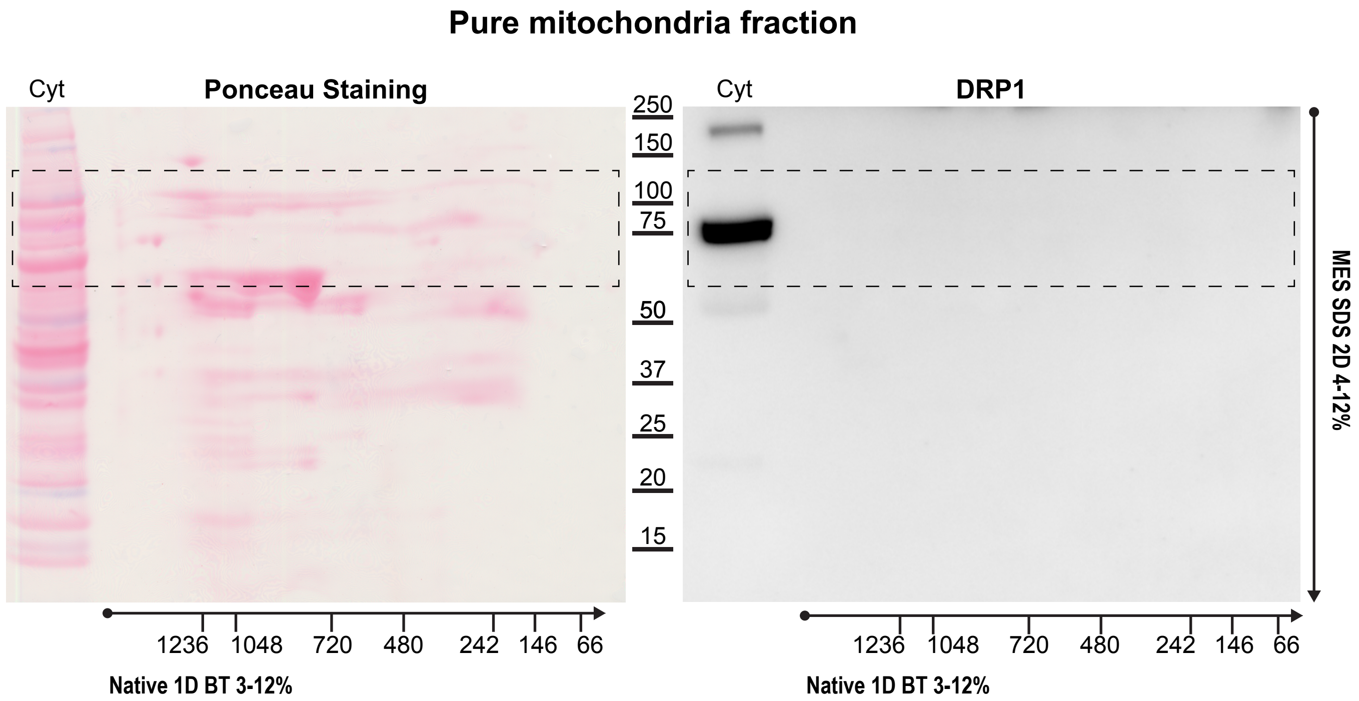

Figure S6. Solubilized proteins from the cytosol, MAM and SR subcellular fractions were separated according to their masses on a linear 3%-12% acrylamide gradient gel for BN-PAGE (first dimension, 1D). Native protein complexes were separated by 4%-12% Bis-Tris SDS-PAGE gradient gel (second dimension, 2D).

Supplementary Figure S7

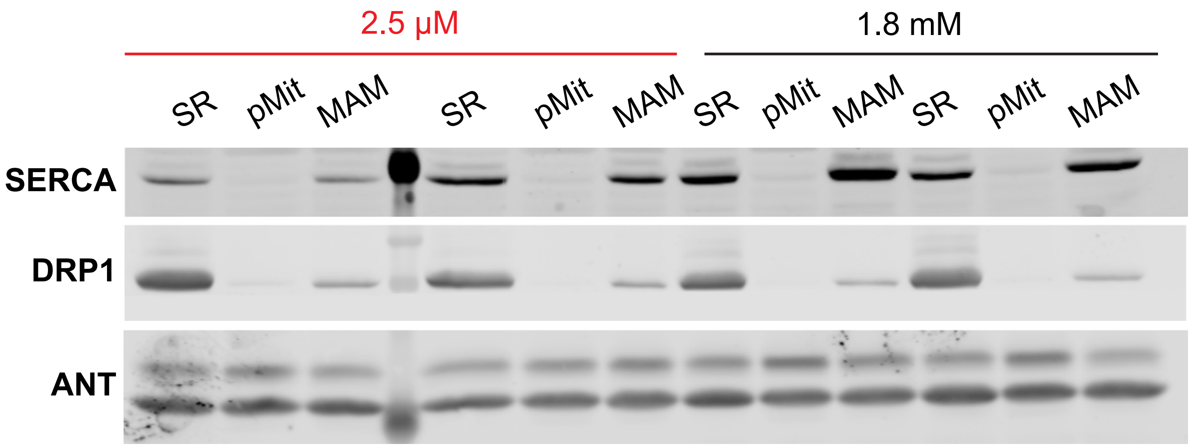

Figure S7. In rat hearts perfused with 2.5μM Ca^{2+} or 1.8mM Ca2+ the DRP1 decrease in SR and MAM fractions was not related with DRP1 presence in the pMito fraction.

Supplementary Figure S8

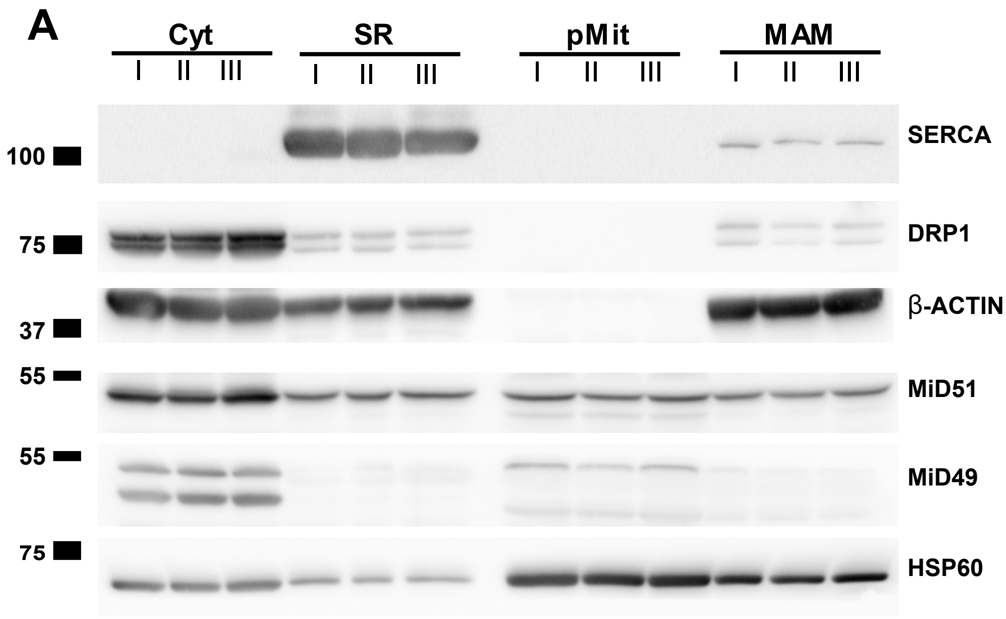

Figure S8. Representative WB of DRP1 proposed anchoring proteins – β-ACTIN, MiD49 and MiD51- showing ditribution among the different cellular fractions (Cyt, SR, pMit and MAM, N=5 animals per fractionation, 3 different fractionations) SERCA, HSP60 and DRP1 were used as quality control of the fracitionation process.

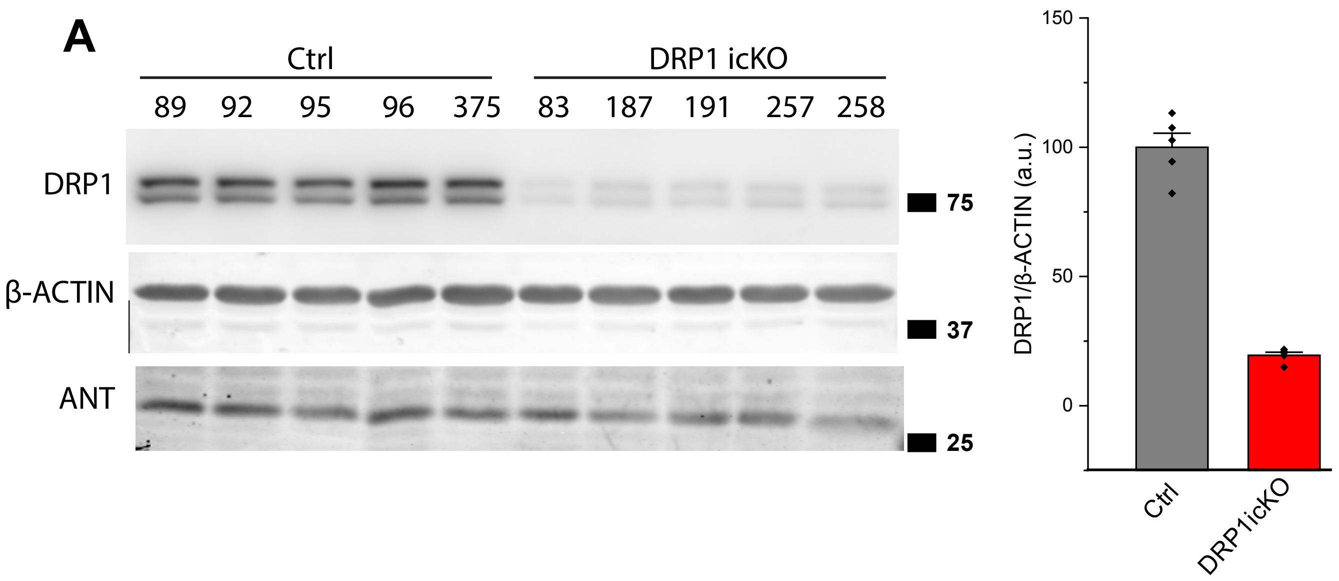

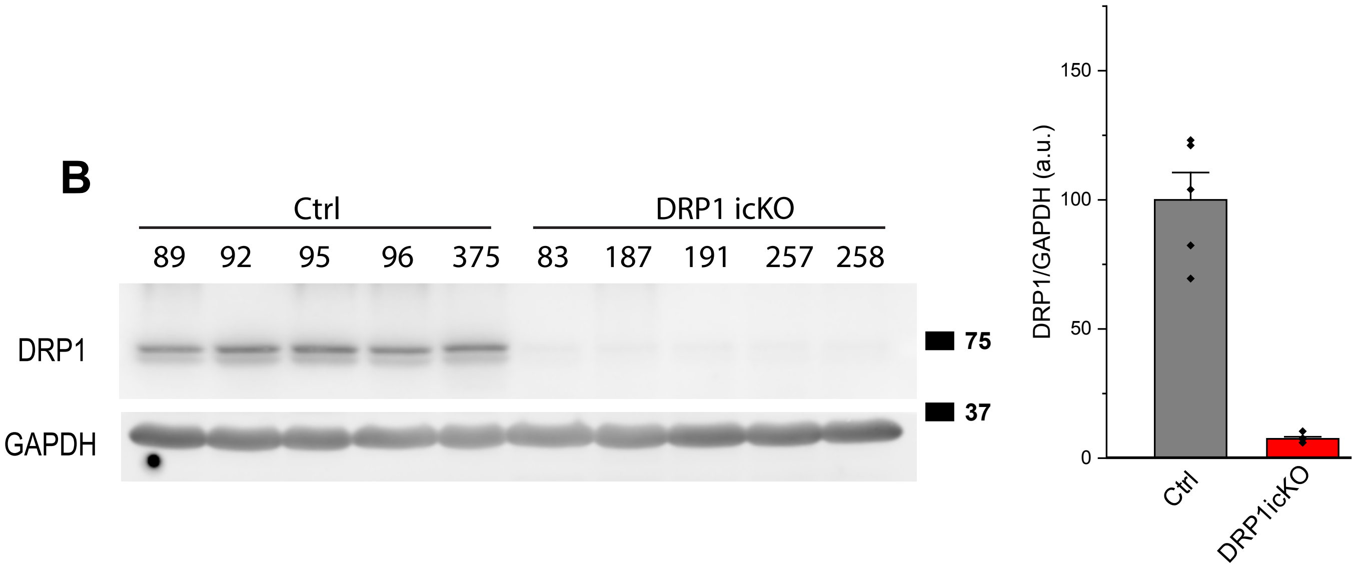

Figure S9. WB analysis and quantification of DRP1 levels of the control and Drp1icko animals in A) Total homogenate and B) Cytosolic fraction (each number corresponds to a single mice, N=5 per group).

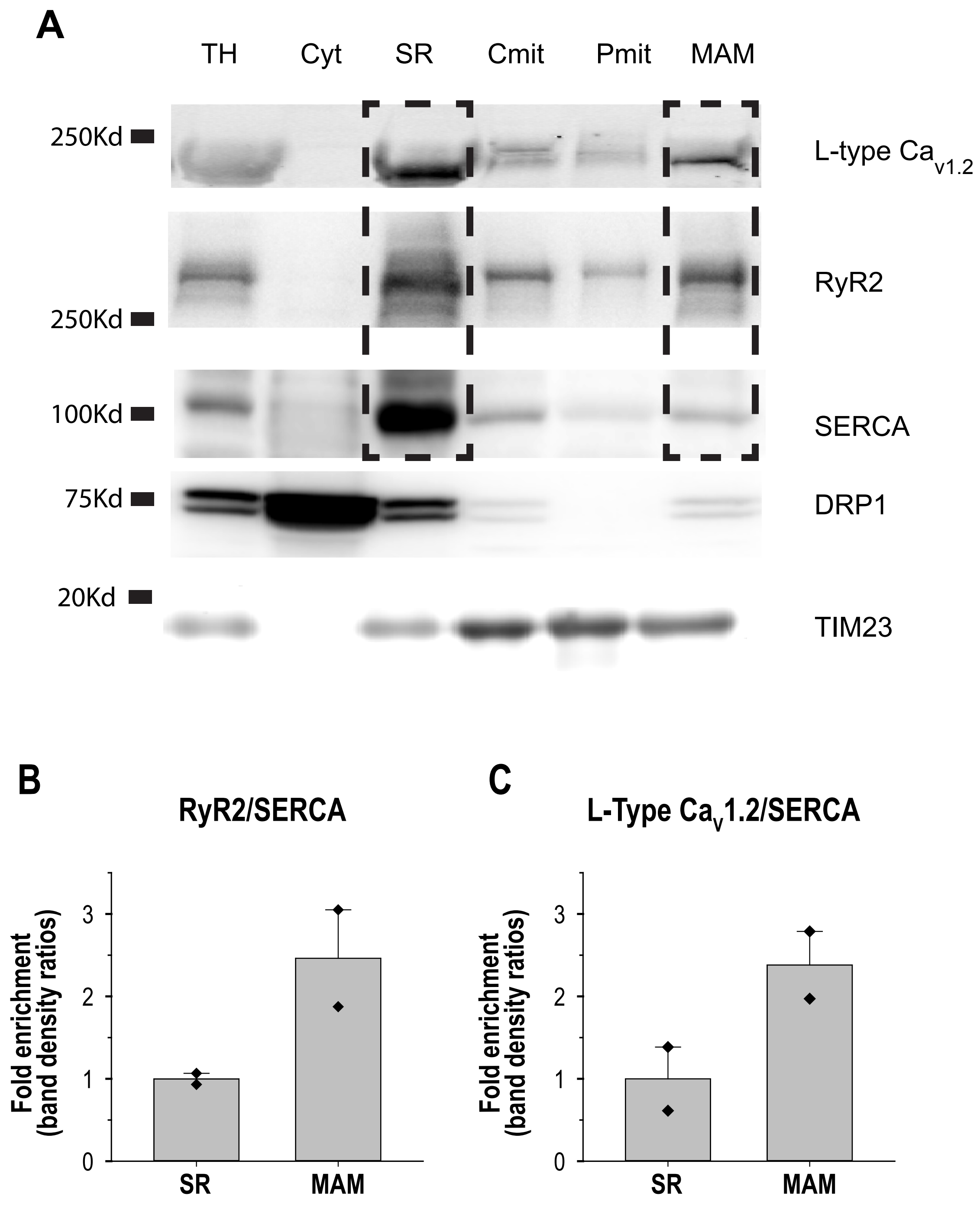

Figure S10. A) Representative Western blot of total cardiac homogenate (TH) and cardiac cellular fractions obtained by differential centrifugation, cytosol (Cyt), sarcoplasmic reticulum (SR), crude mitochondria (cMit) pure mitochondria (pMit) and mitochondria-associated membranes (MAM). RyR2 and L-type Ca2+ channels were used as junctional SR markers while SERCA was used as network SR marker. B-C) Comparison of relative abundance of junctional SR and network SR proteins between SR and MAM fractions. N = 3 fractionations from 5 mice hearts each.
